# Embryonic Phase Transition/Separation on Hepatic Liquid Crystal Droplets Is Essential for Liver Development

**DOI:** 10.3390/biology15141168

**Published:** 2026-07-16

**Authors:** Qinchun Duan, Xixi Cao, Xinjie Li, Zutong Zhang, Yuanlin Miao, Tingting Zhang, Yuane Hou, Boling He, Xin Zhou, Odell D. Jones, Jiali Li, MengMeng Xu, Yingli Liu, Xuehong Xu

**Affiliations:** 1 Laboratory of Cell Biology, Genetics and Developmental Biology, College of Life Sciences, Shaanxi Normal University, Xi’an 710062, China; duanqinchun2020@snnu.edu.cn (Q.D.); caoxixi2020@snnu.edu.cn (X.C.);; 2 Shaanxi Key Laboratory of Ischemic Cardiovascular Disease, Shaanxi Key Laboratory of Brain Disorders, Institute of Basic & Translational Medicine, Xi’an Medical University, Xi’an 710021, China; 3 University Laboratory Animal Resources (ULAR), School of Medicine, University of Pennsylvania, Philadelphia, PA 19104, USA; 4 Department of Internal Medicine, University Hospital, Shaanxi Normal University, Xi’an 710062, China; 5 Department of Pediatrics, Columbia University, New York, NY 10032, USA

**Keywords:** embryonic phase transition (in vivo phase transition), in-drop embryonic phase separation, hepatic liquid crystal droplet (HLCD), LC3A, Beclin 1, birefringent hepatic Maltese crosses, Taihe black-bone silky fowl (*Gallus gallus domesticus* Brisson)

## Abstract

Phase transition or phase separation occurs in cells and tissues in many physiological events, such as protein granules (P granules) in early *C. elegans* development. Inappropriate phase transition is often associated with pathologic processes such as RNA–protein complex (RNP) and FUS-mutation-associated diseases. Given its ubiquity, phase transition has been considered a new frontier for comprehending physiological processes and pathological diseases. However, molecular and cellular mechanisms of phase transition in situ remain poorly described. Combining histochemistry with polarization analysis and biochemical thin-layer chromatography, we identified a massive phase transition change during the development of Taihe fowl (*Gallus gallus domesticus* Brisson). During embryonic day 12 (E12), the livers of these Silkie chicken demonstrated a sudden massive transition from hepatic lipid droplets (HLD) into hepatic liquid crystal droplets (HLCDs). We identified these changes by characterizing the sudden appearance of birefringent Maltese crosses (MCs) typical to liquid crystals (LC) where non-birefringent lipid droplets used to reside within hepatic cells. LC status was confirmed by fluidity with shape-changing and in vitro thermal phase transition tests. These HLCDs were present consistently until the early postnatal days after hatching. Using thin-layer chromatography combined with X-ray diffraction analysis, we determined that these HLCDs were composed of cholesterol, cholesterol ester and lecithin, which are the same as the components of cytoplasmic membrane. There was no change in the quantity of lipid components during liver development to suggest a critical mass of components triggering these changes. However, expressions of membrane-associated autophagy markers LC3A and Beclin 1 increased dramatically during this HLD to HLCD transition. Increases in membrane-associated LC3A and Beclin 1 are localized with massive increases in membrane lipid components of HLCDs. Areas with enhanced LC3A and Beclin 1 signaling have been associated with liquid crystal MCs to the thickness of 69 Å (Bragg d value). These associations indicate the possible regulatory role autophagy plays during liquid crystal phase transition in embryonic liver development. Reactivation of this autophagy pathway may be a possible mechanism behind the development of non-alcoholic fatty liver disease in adulthood.

## Simple Summary

Phase transition/separation plays a critical role in both physiological and pathological processes, yet its molecular mechanisms remain poorly defined. In this study, we investigated hepatic development in chicken Taihe fowl and discovered a striking transition/separation during embryonic development, when hepatic lipid droplets (HLDs) converted into liquid crystal droplets (HLCDs). This transformation was identified by birefringent Maltese crosses, confirmed through polarization analysis, thermal phase transition assays, and biochemical characterization. Consistent with cytoplasmic membrane components, HLCDs persisted into early postnatal stages and were composed of cholesterol, cholesterol ester, and lecithin. Importantly, expression of autophagy-related proteins, Beclin 1 and MAP1LC3A, increased markedly during this transition. This suggests autophagy may help regulate lipid remodeling and liquid crystal formation in embryonic liver development. This discovery may suggest a reactivation of autophagy pathways as a possible mechanism behind the development of non-alcoholic fatty liver disease.

## 1. Introduction

Since functions were unveiled in microtubule assembly, organization, and re-organization in axons and dendrites, phase transition has become an important issue in biological system [1,2]. Transition dynamics from GTP/GDP-tubulin dimers, composed of alpha-tubulin and beta-tubulin, to microtubule organelle drives formation of cell polarity in neuronal development and maturation of dendrites [1,2]. This cytoplasmic phase transition between polymerization and de-polymerization requires cooperation between the Golgi apparatus and rough endoplasmic reticulum [3]. Phase transition or phase reparation has also been shown to play a role in multiple cellular processes, including the formation of protein granules or P granules in early stage of *C. elegans* larva development [4]. A certain concentration of P granules results in automatic phase transition, leading to an accelerated chain-reaction like cytoplasmic reaction where P granules aggregate [1,2,5]. This domino-like physical–biochemical phenomenon described in early *C. elegans* blastocyst cytoplasm may be present in other animals as well. Chain-reaction fusion events have been observed in human stem-cell differentiation systems in vitro [6,7]. Phase transitions have also been reported in hepatic cells from both NAFLD mouse models and human patients [8]. In these systems, liquid crystal-like droplets undergo repeated fusion and extrusion events, while hepatocytes activate LC3A-mediated lipophagy during high-fat diet-induced NAFLD [8].

Recently, liquid crystal (LC)-like drop-tubule formation was discovered in embryoid body (EB) derived from human stem H1 cells (hSC) [6]. Human embryoid bodies developed water drop-tubule-like structures extending from the EB surface at 200 µm intervals towards the center of the hSC-EB. This network, hypothesized to behave as a pre-vascular structure, is distributed throughout the hSC-EB and exhibited liquid crystal properties [6]. Physical forces, such as capillary action, in these LC structures may function as pathways for molecular movement prior to former mammalian angiogenesis. Similarly birefringent droplets under polarization microscope were reported in hepatic cells during development of chicken embryo [7,9]. These droplets were composed of cytoplasmic membrane components—cholesterol, cholesterol ester and lecithin. These major components of the cytoplasmic membrane are essential for providing classic fluid structure membrane structure [10]. This liquidity is transferred into these birefringent hepatic liquid crystal (HLC) droplets (HLCDs) distributed through the hepatocytes [7].

Despite its prevalence during embryogenesis, these massive HLCDs cannot be observed in chick liver after hatching, implying HLCD plays a specific role during liver development and becomes obsolete after. The regulators of HLCD during liver organogenesis remain unknown. We hypothesize autophagy genes, such as MAP1LC3A and Beclin 1, are essential for HLCD in the developing chick liver because autophagy is essential for the regulation of the cytoplasmic membrane lipid components that make up HLCD.

Liquid crystals have been created in vitro by Robinson and Frank using a self-assembling system composed of cholesterol and phospholipids [11,12]. Kurik and Lavrentovich subsequently identified phase transitions within these systems [13,14]. More recently, two independent studies described a spectrum of structural states exhibited by cholesteric liquid crystal droplets [15,16], providing strong evidence for the biological significance of liquid crystal organization and substantially advancing our understanding of the internal structure of embryonic HLCDs in vivo [17,18]. Consistent with our previous observations in avian models, embryonic phase transitions during liver organogenesis occur across multiple bird species, including pigeons, Leghorn chickens, and ducks [11,12], although intra-droplet phase transitions were not recognized at that time.

In order to determine the function of phase transition in HLCD within development, we focus on embryonic phase transition (from isotropic to anisotropic) in the developing liver. We focus on characterizing the initial formation of anisotropic HLCD from isotropic hepatic lipid droplets. These droplets made of cholesterol, cholesterol ester, and lecithin may yield understanding of how fat is stored and processed in the liver. Our data could broaden our fundamental understanding of liquid crystal droplets in embryonic liver development to strengthen our understanding of the role liquid crystals play in mammalian development. This could also provide insight on the development of nonalcoholic fatty liver disease (NAFLD), which is characterized by buildup of intrahepatocyte lipids components consistent with those seen in HLCDs.

## 2. Materials and Methods

### 2.1. Animal Maintenance

Chicken embryos of Taihe black-bone silky fowl (*Gallus gallus domesticus* Brisson) for all experiments were obtained from hatching eggs (Nanchang, China). Fertilized chicken eggs were incubated in Agri-Mech Incubator (Weiqian Agriculture Mechanics Export & Import Ltd. at Dezhou, Shandong, China) under standard conditions at a constant temperature of 37.8 °C and humidity of 60–65%, with egg turning-over every two hours and hatching rate of over 95%. At incubation days of 12th, 14th, 16th, 18th and 20th, the corresponding embryos (E) of E12, E14, E16, E18 and E20 were obtained (N > 8, at each time point). Postnatal (P) samples were collected after eggs hatched at P0 and P2. Dissection was carried out to harvest tissues for subsequent experiments. All animal maintenance and experiment procedures in this study were conducted according to the principles of the Declaration of Helsinki, and the study protocol was approved by the Institutional Ethics Committee of the Shaanxi Normal University (SNNU-CLS2022023).

### 2.2. Sample Preparation and Histological Analysis

Fresh livers dissected from the embryonic chickens (the Taihe black-bone silky fowl) were used for paraffin section and frozen section. For paraffin section, tissues were fixed with 4% paraformaldehyde (PFA) and followed with dehydration with gradient alcohol. Samples were then embedded in paraffin after xylene treatment. Embedded samples were cut to sections with thickness of 10 μm. For frozen section, tissues were placed in a matrix embedding agent (OCT, optimum cutting temperature; Sakura Finetechnical, Ltd., Tokyo, Japan) and immediately frozen. Thickness of cryo-section was set at 12–14 μm. The section collections were carried out at the range of series sectioning for the entire sample. Both frozen and paraffin sections were analyzed for histochemistry, including Hematoxylin and Eosin staining (H&E) and Oil Red staining.

### 2.3. Polarization Microscopy, Phase Transition and Fluidity Analysis

Images captured in experiment under normal light and polarized light were documented with inverted microscopy (Carl Zeiss Microscopy GmbH, Carl-Zeiss-Promenade 10, Jena, Germany) and ZEN core 3.9 software (Carl Zeiss Microscopy GmbH, Jena, Germany). Samples for observation, imagining, and study were obtained under microscope from fresh Taihe chicken liver. For polarization microscopy, samples were placed between glass-slide and cover-slide within PBS (PH7.4) at physiological state for analysis of thermal phase transition and fluidity.

Analyses on in vitro phase transitions were carried out under polarization microscopy as previously described [7,8,19]. Phase transition refers to transition of a substance from anisotropic to isotropic state when the temperature is increased. Liquid crystal and crystal states show optical activity with anisotropic texture with behavior of birefringence. To identify whether the anisotropic texture belongs to either crystalline or liquid-crystalline, their fluidity was inspected while simultaneously measuring their optical activity. Birefringent anisotropic substances that retained fluidity, as confirmed by shape-changing under pressure, were characterized as liquid crystal.

To evaluate fluidity, pressure-and-release (P&R) experiment was applied on Taihe samples from different embryonic livers as previously described [7,8,19]. After P&R was applied, if inspected birefringent substances could continuously change shape, such as fusion/merging between two birefringent MCs, division of one birefringent particle into two or more, blending/amalgamation as one birefringent MCs or tube, etc., these birefringent substances were considered as liquid crystalline. If an inspected birefringent substance was broken into smaller pieces and these pieces could not reform or resume, the birefringent substance was considered crystal.

### 2.4. X-Ray Diffraction and Scattering Analysis

To characterize liquid crystals and crystals in livers during development of Taihe fowl embryos, we used Small-Angle X-ray Scattering (SAXS) and X-ray Diffraction (XRD) to document diffraction patterns of liver samples collected as previously described. SAXS was carried out on a small-angle goniometer of Bruker D8 Advance diffractometer (Bruker AXS GmbH, Karlsruhe, Germany) at diffraction angles (2θ) between 0.2° and 10° with divergence slit (DS) of 0.2 MM. XRD (Bruker AXS GmbH, Karlsruhe, Germany) were documented on a wide-angle goniometer at a diffraction angle (2θ) between 3° and 45° with DS slit of 0.6 MM. All diffractometer experiments were carried out with a copper target, and the working power was set at 1.6 kW (40 kV, 40 mA). Software of MDI Jade 6 was used for XRD data processing.

### 2.5. Thin-Layer Chromatography

Silica plates were used (Qingdao Ocean-chemical Company, Qingdao, China) for thin-layer chromatography (TLC). Fresh tissues of Taihe chicken livers were harvested from embryos at E16, E18 and postnatal newborn chicks P0 and P2. Two-fold volumes of PBS (pH 7.4) were added to homogenize tissues. The mix was then centrifuged at 12,200 rpm for 15 min. After centrifugation, supernatants were collected and dried in a vacuum chamber for overnight or a longer period of time. Before continuing experiment, a chloroform and methanol mixture (1:2 by volume) was added into samples and vortexed vigorously for 2 min. Subsequently, one part chloroform was added, followed by adding one part water, and vortexed vigorously. Five minutes later, samples were centrifuged at 12,200 rpm for 15 min. The denser liquid layer was collected. Two extra centrifugations were carried out for extraction at a temperature of 15 °C.

Equal amounts of each sample prepared as above were dissolved in a chloroform: methanol (1:1 by volume) mixture and loaded in series on a single silica plate 1 cm apart, keeping no more than 2 cm away from both edges. Cholesterol (Sinopharm Chemical Regent Co., Ltd., Shanghai, China), cholesterol ester (cholesteryl oleate, Alfa Aesar Chemicals Co. Ltd., Shanghai, China), and lecithin (Sinopharm Chemical Regent Co., Ltd., Shanghai, China) were set as standards in the line as well. A mixture of chloroform, methanol and water (65:17:1) was used as mobile phase. The final developing pattern of samples with three standards in line was recorded under 245 nm UV light.

### 2.6. Immunofluorescence Analysis

Immunofluorescence staining specific for targeting proteins was performed on paraffin sections of Taihe embryonic liver tissues at different developmental stages (E12, E14, E16, E18 and E20). After blocking with 1% BSA, deparaffinized sections were steamed for antigen activation and then incubated with the primary antibodies before secondary antibodies were applied for fluorescence visualization. Primary antibodies against LC3A (1:50) and Beclin 1 (1:100) were purchased from Abcam, Cambridge, UK. Secondary antibodies (1:50, Jackson ImmunoResearch Lab, West Grove, PA, USA) were used to report first antibody distribution with fluorescence, i.e., Fluorescein (FITC) affiniPure goat anti-mouse IgG (1:50) and Rhodamine Red™-X (RRX) AffiniPure goat anti-rabbit IgG (1:50). Glycoproteins on hepatic cell plasma membrane were labeled with fluorescent Wheat Germ Agglutinin (WGA) (ThermalFisher Scientific, Waltham, MA, USA). Nuclei were visualized with Hoechst 33342 (Sigma–Aldrich LLC, St. Louis, MO, USA) counterstaining. Immunofluorescence images were recorded by Inverted microscopy Zeiss Observer A1 microscope (Carl Zeiss Microscopy GmbH, Carl-Zeiss-Promenade 10, Jena, Germany), and image analysis was performed using manufacture ZEN software.

### 2.7. Western Blot for Expression Analysis

Taihe embryonic and postnatal livers were harvested at E16, E18, E20 and E22. Samples of E12 and E14 were attempted but, due to liver size, provided minimal HLCD samples. Samples were homogenized in RIPA lysis buffer with protease inhibitors (TargetMol, Shanghai, China) on ice. Centrifuging at a speed of 1000 rpm for 10 min was applied and HLCDs were collected from the top layers as previously described [20]. Total protein levels were quantified by using the BCA assay from the HLCD top layers (Beyotime, Shanghai, China). Equal-quantity loaded samples (20 μg for each load) were separated by sodium dodecyl-sulfate polyacrylamide gel electrophoresis (SDS-PAGE). Here, 5% and 10% gels were used as stacking and separating gel individually. Samples on gel were transferred to PVDF membranes and blocked with 5% non-fat dried milk. After washing with TBS-T, they were incubated with primary antibodies overnight with constant oscillation at 4 °C and subsequently incubated with appropriate horseradish peroxidase (HRP)-conjugated secondary antibodies. Protein blotting documentation was detected with chemo-luminescence system (Tanon Fine Do X6+ Multi, Shanghai, China).

Primary antibodies LC3A, Beclin 1, MMP2 and MMP8 were purchased from Abcam (Abcam, Cambridge, UK); FKBP12, FKBP14 and FKBP52 antibodies were purchased from Novus (Littleton, CO, USA); Integrin αV antibody was purchased from BD biosciences (San Jose, CA, USA); GAPDH antibody was purchased from Proteintch (Chicago, IL, USA). HRP-conjugated goat anti-mouse IgG and goat anti-rabbit IgG secondary antibodies were purchased from Jackson ImmunoResearch Lab Incorporation (1:10,000; West Grove, PA, USA).

### 2.8. GepLiver Database Analysis

The GepLiver database (http://www.gepliver.org/; accessed on 8 January 2026) is a longitudinal and multidimensional atlas of liver gene expression. It integrates bulk RNA sequencing data from 2469 human, 492 mouse, and 347 human samples. It contains single-cell expression profiles of 409,775 cells and data from 27 hepatocyte cell lines, collectively covering 16 distinct liver phenotypes. Using the RNA-seq data retrieved from this resource, quantitative analysis and normalization were performed and followed by a comparative assessment of the expression levels of BECN1 and LC3A in normal versus non-alcoholic fatty liver disease (NAFLD) tissues. Gene expression values were log_2_ (TPM)-transformed, and statistical significance was defined as *p* < 0.05 [21,22]. We obtained single-cell RNA-seq (scRNA-seq) data from the same database. Cells were visualized in low-dimensional space using Uniform Manifold Approximation and Projection (UMAP) and were classified into major hepatic and immune populations—including hepatocytes, cholangiocytes, CD4^+^ T cells, CD8^+^ T cells, non-conventional T cells, NK-like cells, B cells, plasma cells, monocytes, macrophages, dendritic cells, neutrophils, mast cells, endothelial cells, fibroblasts, and erythroid cells—based on established canonical markers. Finally, UMAP-based visualization was performed to examine BECN1 expression across these annotated cell clusters [21].

### 2.9. STRING Database Analysis

The STRING database (https://string-db.org/, accessed on 12 January 2026) is a comprehensive resource integrating known and predicted protein–protein interactions. It consolidates experimental data, text-mined evidence from PubMed and other literature, and computational predictions across more than 5000 organisms, encompassing approximately 24.6 million proteins. This platform further supports functional enrichment analysis of gene sets. Using the feature genes of the fetal cells provided in GepLiver, protein–protein interaction (PPI) analyses with BECN1 and LC3A were conducted. Proteins that directly interact with each of these two proteins were subsequently screened for PPI network construction.

### 2.10. Statistics

Image analysis software of ImageJ 1.54 (National Institutes of Health, Bethesda, MD, USA) was used to analyze the size, birefringence intensity, signals of immunofluorescence staining and HRPase productions, etc., which were documented for our experimental subjects at different developmental stages of Taihe fowl. Statistical analysis was performed by SPSS29.0 (IBM, Armonk, NY, USA) and then graphed using GraphPad Prism 8 (GraphPad Software, La Jolla, CA, USA). The data obtained by DSC were analyzed and graphed using OriginPro8.5 (OriginLab Corporation, Northampton, MA, USA).

## 3. Results

In chicken development, liquid-crystal droplets in the form of Maltese cross-shape birefringence were discovered in the developing heart, lung, kidney, brain, and yolk sac [7,19]. The birefringence appearance and phase transition properties of liquid crystals were used to describe HLCD in vivo in the embryonic liver of Taihe chicken embryo. Characterization of HLCDs components was also performed. HLCDs were found to be composed of cytoplasmic membrane components. Autophagy genes encoding LC3A and Beclin 1 proteins were found to be associated with formation of HLCD.

### 3.1. In Vivo Phase Transition to the Liquid Crystal Droplets Occurs in the Embryonic Developing Liver

During development of Taihe chicken embryo, we found that hepatic cytoplasmic droplets underwent an in vivo phase transition from non-birefringent isotropic state to birefringent anisotropic state as defined by the presence of Maltese cross birefringence. This embryonic phase transition is considered as a unique phenomenon critical for the development of embryonic liver. This embryonic phase transition could be found in many other tissues and organs where HLCDs have also been reported [7,19]. Here, we focused on embryonic phase transition in developing liver of Taihe fowl embryo.

**Figure 1:**
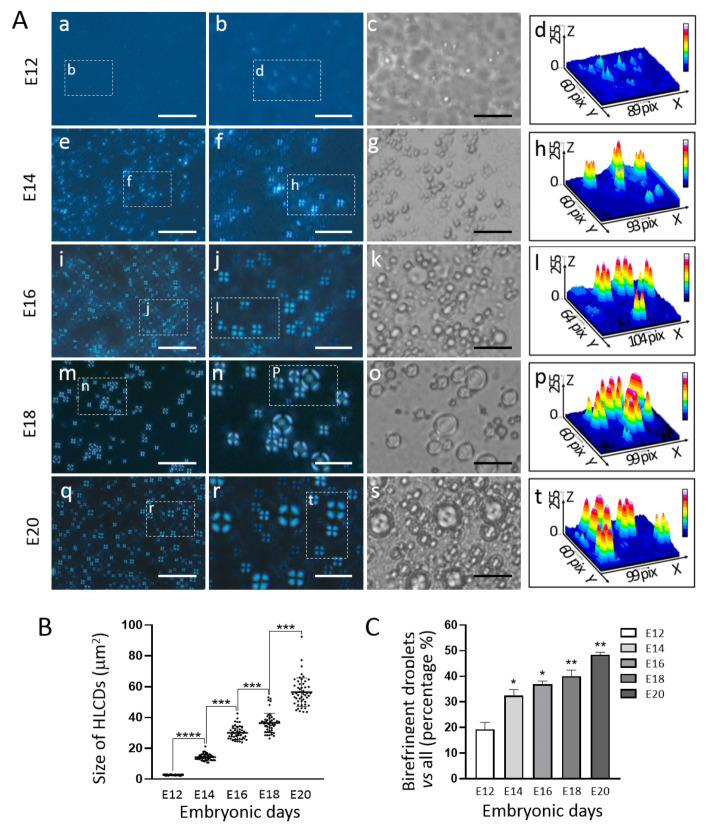
Characterization of hepatic birefringent droplets in form of Maltese crosses during development of embryonic livers of Taihe silky fowl at different stages of chicken embryo development. (Panel (**A**) (**a**,**b**,**e**,**f**,**i**,**j**,**m**,**n**,**q**,**r**)) are images of hepatic Maltese crosses in livers at E12, E14, E16, E18 and E20 under crossed polarizers respectively. (Panel (**A**) (**b**,**f**,**j**,**n**,**r**)) are enlarged from (Panel (**A**) (**a**,**e**,**i**,**m**,**q**)). (Panel (**A**) (**c**,**g**,**k**,**o**,**s**)) are their corresponding images under non-crossed polarizers. (Panel (**A**) (**d**,**h**,**l**,**p**,**t**)) show density of birefringent droplets in certain zone of (Panel (**A**) (**b**,**f**,**j**,**n**,**r**)). Panel (**B**) is statistics analysis on size increase of hepatic birefringent droplets; Panel (**C**) is statistics analysis on number increase of hepatic birefringent droplets (number of birefringent particles vs. number of all particles including birefringent droplets and non-birefringent droplets at E12, E14, E16, E18 and E20 along with embryonic development. Scale bars are 50 μm in (**a**,**e**,**i**,**m**,**q**); Scale bars, 15 μm in (**b**,**f**,**j**,**n**,**r**,**c**,**g**,**k**,**o**,**s**). (* *p* < 0.05, ** *p* < 0.01, *** *p* < 0.001, **** *p* < 0.0001).

We systematically inspected the timeline of HLCD development in the developing liver of Taihe fowl using polarization microscopy. Birefringent droplets were observed in the developing liver at developing stage of day E12. These LC phase droplets gradually increased on hepatic cord (Figure 1A) and peaked at E20 (Figure 1B). This coincides with peak fluid flow in the hepatic sinus (time lapse recording presented in Figure 7). After E20, the LC droplets maintained their size and density within hepatic cells until postnatal days (P) 12–14, well after hatching.

During the embryonic phase transition (Ph_iso-LD→ani-HLCD_) at E12, the individual HLCD grew in size with Maltese cross texture from size about 3 μm^2^ at E12, to 14 μm^2^ at 14, 30 μm^2^ at 16, 36 μm^2^ at E18 and 56 μm^2^ at E20 maximally (all *p* < 0.0001; Figure 1B). HLCDs continuously multiplied after the initiation of Ph_iso-LD→ani-HLCD_ at E12. This can be seen in the increase in number and percentage of Maltese cross HLCDs vs. total non-birefringent liquid droplets in each embryonic day henceforth (E14 vs. E12, *p* < 0.05; E16 vs. E14, *p* < 0.05; E18 vs. E16, *p* < 0.01; and E20 vs. E18, *p* < 0.01). This change in phase transition (Ph_iso-LD→ani-HLCD_) along with massiveness increase indicates that Ph_iso-LD→ani-HLCD_ observed at E12 must be increasingly necessary for Taihe embryonic development.

### 3.2. The Embryonic Phase Transition/Separation to Liquid Crystal Is Initiated Within Individual Droplets

We performed detailed analysis on LCDs from E16 embryonic liver to further understand the mechanism of the embryonic phase transition. We systematically analyzed the behavior of individual HLCDs and captured a unique in-droplet phase transition, which we defined as in-drop embryonic phase transition.

**Figure 2:**
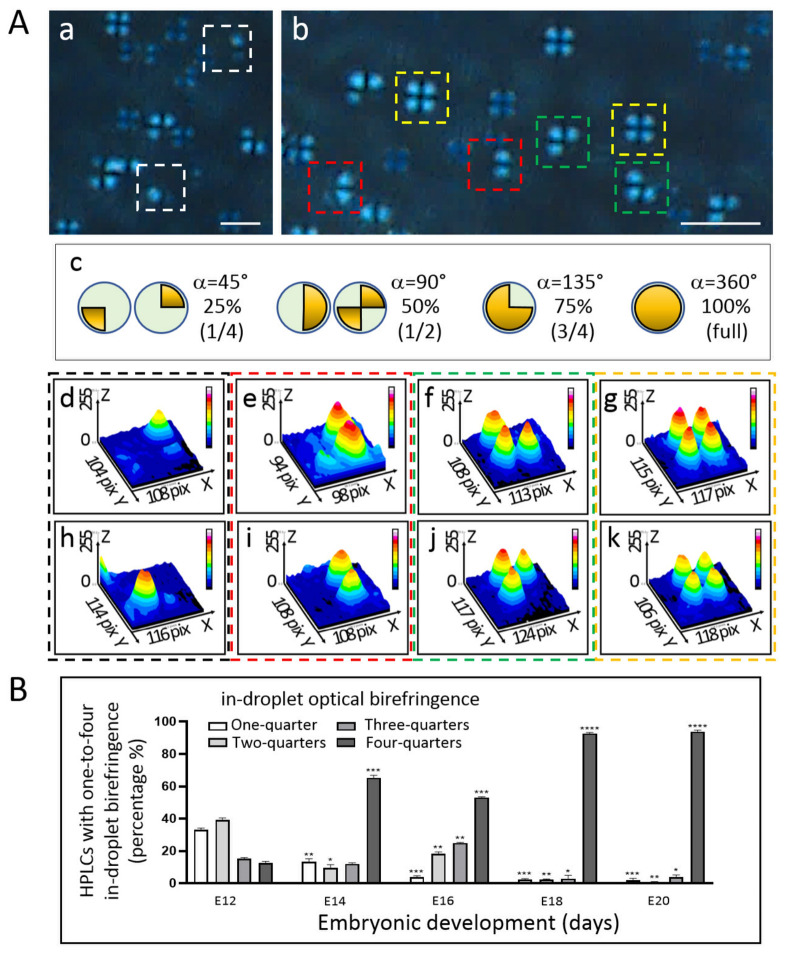
Analysis on optical activity of hepatic birefringent droplets via evaluating birefringence percentage within hepatic Maltese crosses in embryonic livers at E12, E14, E16, E18 and E20. (Panel (**A**) (**a**,**b**)) present two classic views of E16 embryonic livers under crossed polarizers. Scale bars are 5 μm in ((**A**) (**a**)) and 10 μm in ((**A**) (**b**)). Maltese crosses show optical activity with 1/4 (25%) birefringence in hepatic Maltese crosses indicated with broken white-line square corresponding to (Panel (**A**) (**c**,**g**)) highlighted with black broken-line square sketched as (Panel (**A**) (**c**) (left)). Maltese crosses with 1/2 (50%) birefringence, 3/4 (75%) and 100% birefringence in (Panel (**A**) (**b**)) correspond to (Panel (**A**) (**d**–**i**)), sketched as (panel (**A**) (**c**) (midle-left)), (Panel (**f**,**j**)) sketched as (Panel (**A**) (**c**) (midle-right)), and (Panel (**g**,**k**)) sketched as (Panel (**A**) (**c**) (right)) highlighted with red, green and yellow broken-line squares separately. In-drop birefringence increase on optical activity of embryonic liver HLCDs at E12, E14, E16, E18 and E20 is documented in Panel (**B**). Significant increase in full birefringence Maltese crosses is in E14 liver and reaches a maximum in E18 compared to E12 (* *p* < 0.05, ** *p* < 0.01, *** *p* < 0.001, **** *p* < 0.0001, when compared to each item of E12).

In individual hepatic droplets, the phase transition to liquid crystal was observed by optical activity first beginning in a small zone, which we categorized as one-quarter-birefringent droplets (white-line box) of E16 livers (Figure 2A(a) sketched as c (left)). These birefringent zones then enlarged within each droplet with liquid-crystallization from the surface towards the center of droplets to form two-quarter-birefringent droplets (red box, Figure 2A(b) sketched as c (middle-left)), three-quarter-birefringent droplets (green box, Figure 2A(b) sketched as c (middle-right)), and four-quarter-birefringent droplets (yellow box, Figure 2A(b) sketched as c (right)). Within the developing embryo, this liquid-crystallized zone presented as birefringent optical activity in droplet enlarged in a manner of birefringence fanning stripping and within a Maltese cross. The in-droplet birefringence continuously broadens with the fanning stripping unveiled using polarization analysis. This process, which we termed fanning stripping birefringence, presents as birefringence in one portion of the Maltese cross under polarization analysis. Using crossed polarizers, we found this fanning stripping birefringence increased with each day of Taihe development after E12. In liver, 3.9% of the droplets exhibited approximately one-quarter of fanning stripped birefringence (Figure 2A(d,h),B), and 18.2% of the droplets presented with two-quarters of birefringence (Figure 2A(e,i),B). At E18, 24.9% and 53.0% of the droplets possessed three-quarter (Figure 2A(f,j),B) and fully fanning stripped birefringence (Figure 2A(g,k),B), respectively.

At E14 and E16, the droplets with four quarters of birefringence significantly increased up to 65.3% and 53%, respectively (Figure 2A(c)). By E18 and E20, the droplets with four quarters or full birefringence were dominant and reached over 92.6% and 93.8% (Figure 2A(c)). This birefringence activity persistent until P12–P14. This continuous increase in optical birefringence activity in the individual HLCDs along with embryo development indicates that the function of this in-droplet embryonic phase transition along with embryonic phase transition is necessary for liver architecture. We hypothesize that this phase transition to liquid crystal within individual droplets strengthens the developing liver tissue via physical properties inherent to liquid crystals.

### 3.3. Lipid Component Identification of the Hepatic Liquid Crystal Droplets During Taihe Embryonic Development

**Figure 3:**
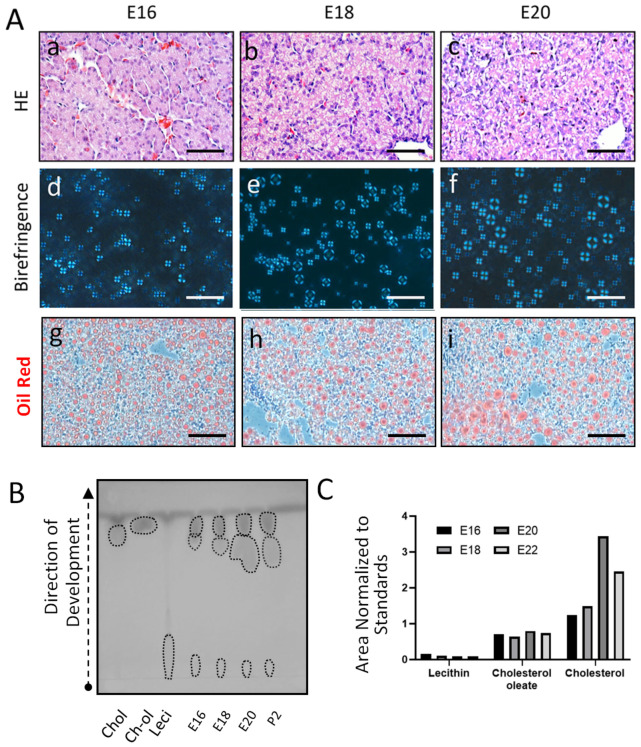
Lipid component identification on of hepatic birefringent droplets with Oil Red and thin layer chromatography in the embryonic livers. Panel (**A**) shows analysis of histology and histochemistry and polarization microscopy on the embryonic livers at E16, E18 and E20. H&E staining for histologic geographic view (Panel (**A**) (**a**–**c**)), optical activity for birefringent Maltese crosses (Panel (**A**) (**d**–**f**)) and Oil Red staining for lipid droplets (Panel (**A**) (**g**–**i**)). Scale bars are 30 μm in (**a**–**i**). Analysis using thin layer chromatography on the extracted HLCDs of E16, E18, E20 and P2, cholesterol, cholesterol ester and lecithin were identified as major components of hepatic birefringent droplets in the embryos (Panels (**B**,**C**)). Dashed-line surrounded zones exhibit corresponding area of lipid components respectively.

Employing histology, histochemistry, and thin-layer chromatography, we identified the lipid components of HLCD in Taihe embryo. These components were further confirmed with Small-Angle X-ray Scattering (SAXS) and X-ray Diffraction (XRD). We performed multiple procedures on the hepatic liquid droplets at different development stages of E16, E18 and E20, including Oil Red staining for histochemistry identification and H&E for histological morphology accompanied by crossed polarizer macroscopy. As we present in Figure 3, the HLCDs were mainly composed of lipids displayed in red-drop shape with Oil Red staining in embryonic livers of E16, E18 and E20 (Figure 3A(d–i)). When Oil Red staining was matched with H&E histological structures, lipid droplets were found to be mostly distributed within hepatocytes along the hepatic cord with sinusoid clearing. Thin-layer chromatography for lipid component analysis on extracted HLCDs from livers at days E16, E18, E20 and P2 showed HLCDs were mainly composed of cholesterol, cholesterol ester and lecithin in samples from all four embryonic stages (Figure 3B,C). Of note, cholesterol and cholesterol ester made up an increasingly greater proportion of HLCD until hatching but remained stable postnatally between E20 and P2 (Figure 3B,C).

**Figure 4:**
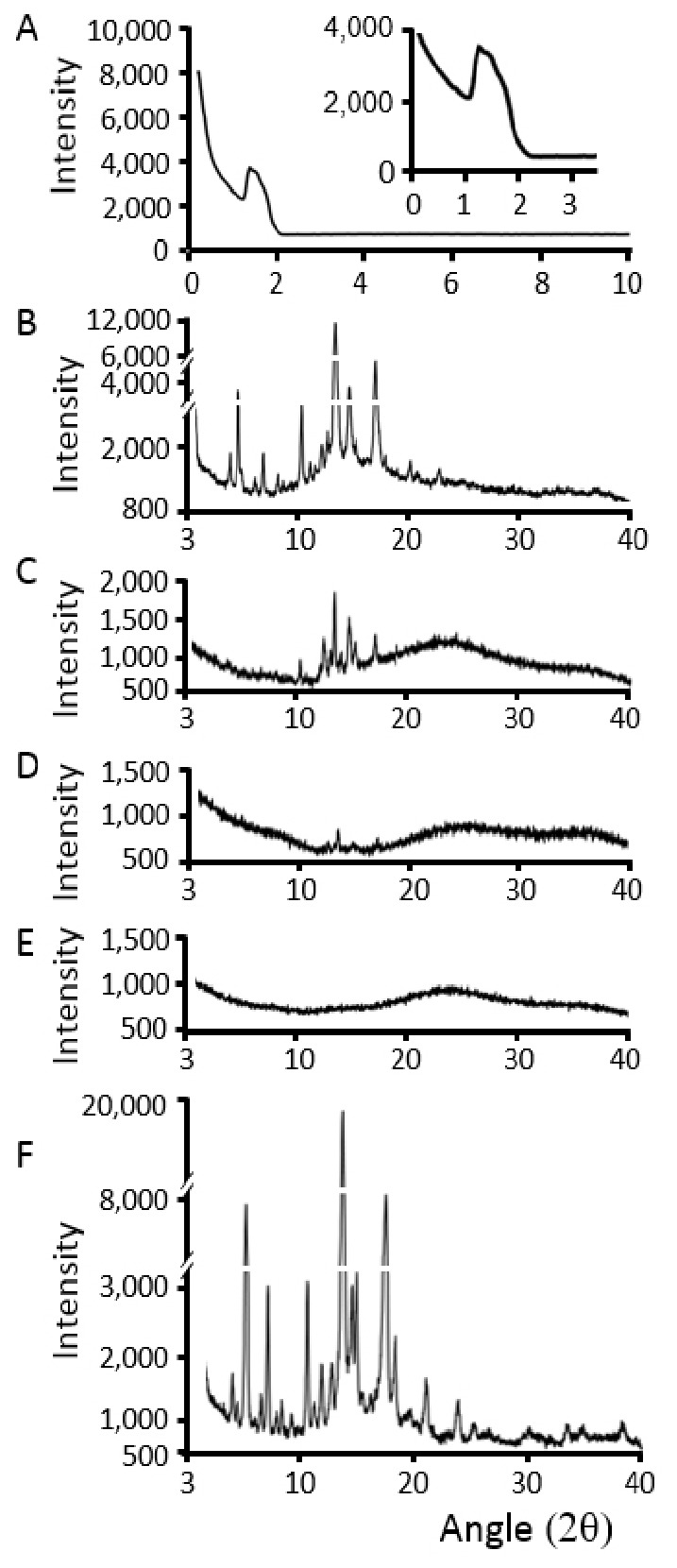
Small-angle X-ray scattering (SAXS) and X-ray diffraction (XRD) characteristics of liquid crystal, crystal and its extracts from the embryonic livers with a diffraction angle range of 3–45° (2θ) and a DS slit of 0.6 MM. Spectrums of sample (**A**) are documented of SAXS and XRD pattern of fresh embryonic liver at E17. Spectrum of sample (**B**) is documented from XRD purified embryonic HLCDs at E20. Samples (**C**,**D**) are XRD patterns of liver tissue of E20 and E17. Samples (**B**–**D**) were stored at −20 °C then thrown to room temperature before inspection. Panel (**E**) is XRD of fresh liver at E17. Panel (**F**) is XRD of standard of cholesterol oleate, which all XRD patterns are compared to. The detailed information is listed in Table 1.

Using previously described methods [7,8,19], SAXS and XRD optical activity of hepatic crystal structures extracted from HLC droplets of Taihe embryonic liver were examined. Sample scattering and diffraction were conducted within the detection spectrums of 0.2° to 10° (Figure 4A and inset) and 3° to 45°, respectively (Figure 4B–E). XRD was carried out on fresh E17 Taihe embryonic liver with no diffraction peak (Figure 4E), and a single peak at lattice planes d(Å) of 69.001 was detected within the spectrums range of 0.2° to 10° degrees, in which the sole peak was documented (Figure 4A and inset). This result solidly classified characteristics of the HLCD. Our data showed that the embryonic phase transition Ph_iso-LD→ani-HLCD_ at E12 is a critical physical event in liver development.

Patterns of XRD spectral peaks from E17 and E20 embryonic livers were compared among the XRD patterns of extracted and fresh HLCD specimens (Figure 4B–E). Normal liver controls showed no diffraction (Figure 4E). Diffraction peaks of HLCD isolated from E20 livers most closely identified with those of cholesterol ester and cholesterol (Figure 4B). Similar findings are reported for E17 (Figure 4D) and E20 livers (Figure 4C). XRD spectrum of the extracted specimens from the embryonic liver of E20 Taihe liver in crystal identified diffractions at lattice planes d(Å) of 8.3364, 7.796, 6.6806, 5.9635, 5.4893, 4.9179, 4.603, 4.0940, 3.9417 and 3.4857 corresponding to I/I0 (11.8), I/I0 (16.1), I/I0 (11.0), I/I0 (24.7), I/I0 (14.5), I/I0 (100), I/I0 (32.2), I/I0 (44.7), I/I0 (16.1) and I/I0 (13.3) in a spectrum range of 5° to 45°, which is consistent with the spectral pattern of cholesterol ester (Table 1 and Figure 4B). Another set of diffractions from embryonic liver of E20 Taihe embryo in crystal was at lattice planes d(Å) of 6.9519, 5.2941, 5.126 and 3.5811 corresponding to I/I0 (12.5), I/I0 (17.6), I/I0 (20.0) and I/I0 (15.3) in the spectrum of 3° to 45°, which was consistent with the spectral pattern of cholesterol (Table 1 and Figure 4B). The strongest XRD diffraction peaks were also observed in the unextracted specimens of the E20 livers (Table 1 and Figure 4C,D), in which the diffraction in the E20 specimen was stronger than that in the E17 specimen. XRD spectrum of E17 Taihe embryonic liver in crystal diffracted at lattice planes d(Å) of 5.199, 4.9335, 4.6034 and 4.1044 corresponding to I/I0 (83.8), I/I0 (100), I/I0 (82.3) and I/I0 (88.2) within the same spectrum range. From this data, HLCD crystals are mainly composed of cholesterol ester and cholesterol. Combining these XRD spectral analyses and thin-layer chromatography data, the lipid components of the HLCDs are cholesterol, cholesterol ester and lecithin.

### 3.4. Fluidity of Taihe Hepatic Liquid Crystal Droplets

Fluidity in conjunction with optic activity are the main properties of liquid crystals commonly used to distinguish them from crystal states. We employed these characteristics to confirm HLCD in birefringent drops of Taihe embryo by demonstrating successful phase transitions from liquid crystal to isotropic lipid droplet to crystal. Using the classic pressure-and-release procedure (P&R) and concomitant optic activity measurements, we confirmed the existence of Taihe embryonic hepatocyte HLCDs.

Previously described cryo-section methods were used on all specimens to retain maximum HLCDs content [7,8,19]. For fresh smeared specimens, P&R experiments were conducted immediately. These cryo-section samples were first heated to 42 °C and then rapidly cooled to approximately 10 °C for 30 to 60 s before resuming room temperature. HLCDs samples confirmed by optic activity and temperature response were placed between polarized prisms (polarizer and analyzer). Prior to pressure application, the birefringent LC-HLDs retained the characteristic four equal-distributing quadrants of birefringence known as classic liquid crystal Maltese crosses. Once P&R was applied, these embryonic HLCDs demonstrated their fluidity with significant shape change (Figure S1A,B). After pressure was released, these elongated HLCDs were able to regain their original shape and classic Maltese cross birefringence (Figure S1 from A(f) in Panel A to B(f) in Panel B). The full-time lapse documenting HLCD response to P&R is recorded in a supplemental figure (Figure S1A(g,h). This described fluidity and birefringence in Taihe HLCDs under P&R confirmed their liquid crystalline properties.

The fluidity of birefringent texture in Taihe HLCDs can also be observed during in vitro thermal phase transition (Figure S2). When HLCD fluidity is increased with heating, two individual HLCDs (Figure S2A,B, MCα and MCβ) are observed fusing together to form a larger MC-HLCD (MC α + β) (Figure S2A,B, MCχ and MCδ). Fusion of two MC-HLCDs (MCα and MCβ was characterized as the sum of size and optical activity of MCα and MCβ (Figure S2B) as calculated from time lapse recording (table in Figure S2B). Shape alternation and fusion of birefringent droplets after P&R are strictly liquid crystal traits. Their existence in developing Taihe hepatocytes confirms the birefringent particles as HLCDs.

### 3.5. In Vitro Thermal Phase Transition of Taihe Hepatic Liquid Crystal Droplets

Understanding characteristics of liquid crystal in biological systems can provide useful information for distinguishing the significance of this physical property and its potential role in the developmental system. We inspected in vitro thermal phase transition of HLCDs from Taihe developing embryos. Our previous work with liquid described a 3-dimensional liquid crystal-like network in stem cell differentiated embryoid bodies. This network seemed to be a precursor to a vascular system needed for nutrient and waste passage in the early embryo [6]. HLCDs have also been identified in NAFLD animal models. These droplets also appear to be liquid crystal in nature [7,8]. There are currently no therapies for NAFLD other than weight and serum lipid management. Better understanding of HLCD may create a therapeutic target for the disease.

In vitro phase transition experiments on HLCD were conducted on smear samples and cryo-sections from E17 Taihe liver. For smear specimens, thermal phase transition was detectable from anisotropic state to the isotropic states (Ph_ani-HLCD→iso-HLD_) and documented with time lapse recording (Figure S3A(a,b),B(a–c,e,f)). In this phase transition, optical activity changed from high birefringent density to low birefringent density (Figure S3B(d–h)). When temperature decreased, the non-birefringent isotropic HLCD underwent reversal phase transition from the isotropic states to anisotropic state (Ph _iso-HLD→ani-HLCD_) (Figure S3A(b,c),B(f,g,i–k)). Here, optical activity resumed from low birefringent density to high (Figure S2B(h,i)), in which the birefringent density of HLCDs recovered back to the original maximal density. During in vitro phase transition, fusion between two individual MCs was also observed (Figure S2).

### 3.6. Dynamic Analysis of Expression and Distribution of Beclin 1 and LC3A in HLCDs via Lipid Component of HLCDs

**Figure 5:**
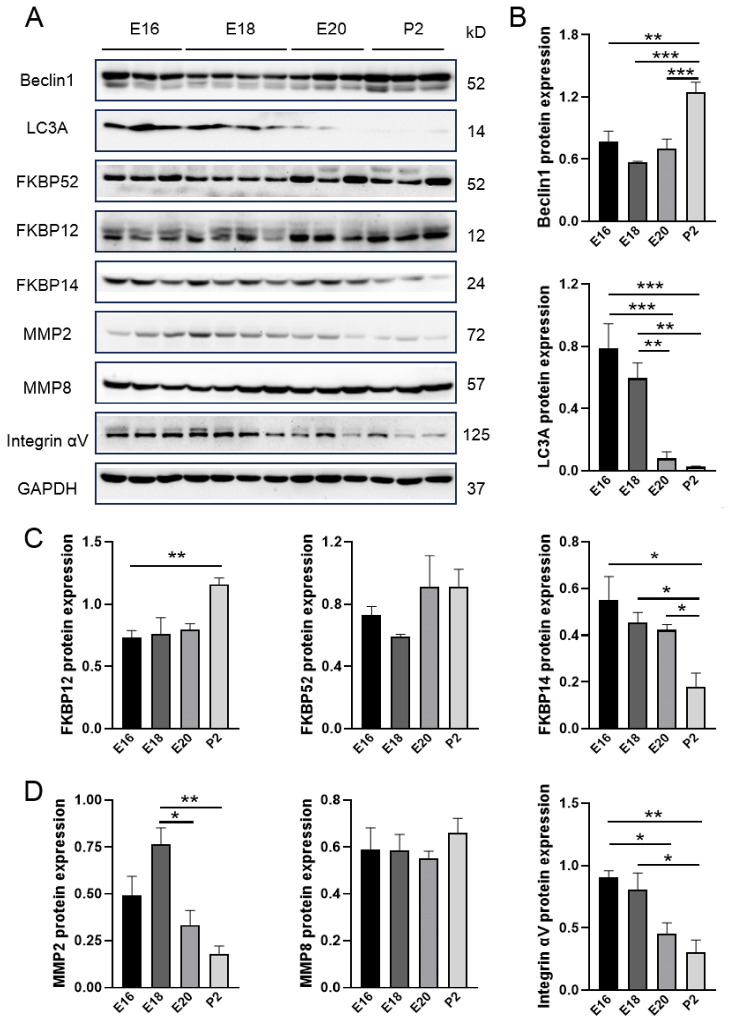
Examination of protein expression of autophagy-related gene, FK506 binding protein (FKBP) gene, and extracellular matrix protein (ECM). Western blotting analysis of autophagy-related proteins, FKBP genes, extracellular matrix proteins (Panel (**A**)) and their statistical analysis of two autophagy related proteins, Beclin 1 and LC3A in Panel (**B**), FKBPs (FKBP52, FKBP12 and FKBP14) in Panel (**C**), and ECM (MMP2, MMP8, and Integrin αV) in Panel (**D**). (* *p* < 0.05, ** *p* < 0.01, and *** *p* < 0.001, normalized to GAPDH.).

Rapid cell membrane building is critical during development and organogenesis. Acquiring the components for cell membrane is a major hurdle for embryonic and tissue growth. Autophagy is a process essential for recycling cellular components to provide such basic building blocks. Beclin 1 is a central scaffolding protein essential for initiating autophagy and recycling of damaged organelles [23,24]. The gene encoding Beclin 1, *BECLIN1*, is a well-recognized regulator of lipids essential for cell biological membrane trafficking processes [25,26]. *BECLIN1* is also a key gene for early embryonic development and cardiac remodeling after ischemia. In post-ischemic hearts, Beclin 1 associates with mitochondria in a specific cytoplasmic zone (mitochondria-associated membranes) to rescue cardiovascular function from endotoxemia [24]. Another critical microtubule-associated (MAP) autophagy protein, LC3A, mediates direct interactions between microtubules and cytoskeleton components. LC3A-II is in charge of autophagosome formation by binding to membrane phospholipid phosphatidylethanolamine (PE, lecithin) [27,28]. Since the three major components of HLCDs are also major components of the cytoplasmic membrane, it stands to reason that autophagy genes such as *BECLIN1* and *LC3A* may also play a role in HLCD formation [29,30]. As mediators of eukaryotic organelle development through membrane-mediated compartmentalization, these genes are already involved in liver architectural development and organization [31,32]. Calcium-related proteins and their regulators have recently shown to be critically important in liver pathogenesis [8,33]. Thus, we carried out systematic investigation of autophagy, calcium-related proteins, and their regulators (i.e., Beclin 1, LC3A, MMPs, and FKBPs) in relation to the formation of HLCDs (Figure 5 and Figure S4). Significant alterations in Beclin 1, LC3A, FKBP12, and Integrin αV expression were found in conjunction with HLCD formation during Taihe fowl hepatogenesis.

Protein expression of HLCD lysates prepared from the livers of Taihe fowl at E16, E18, E20, and P2 were compared via Western blot analysis. Beclin 1 expression was lowest at E18, with a subsequent increase across E20 and P2. The highest level of Beclin expression was at P2. LC3A expression was highest at E16, with a significant decrease at each timepoint thereafter. Levels of LC3A were nearly undetectable by E20. Both FKBP14 and Integrin αV displayed a gradual decline in expression during development. In contrast, MMP2 expression demonstrated an initial rise, peaking at E18, followed by a subsequent decline. The expression levels of FKBP52, FKBP12, and MMP8 did not show any significant trends or differences among the four time points examined.

**Figure 6:**
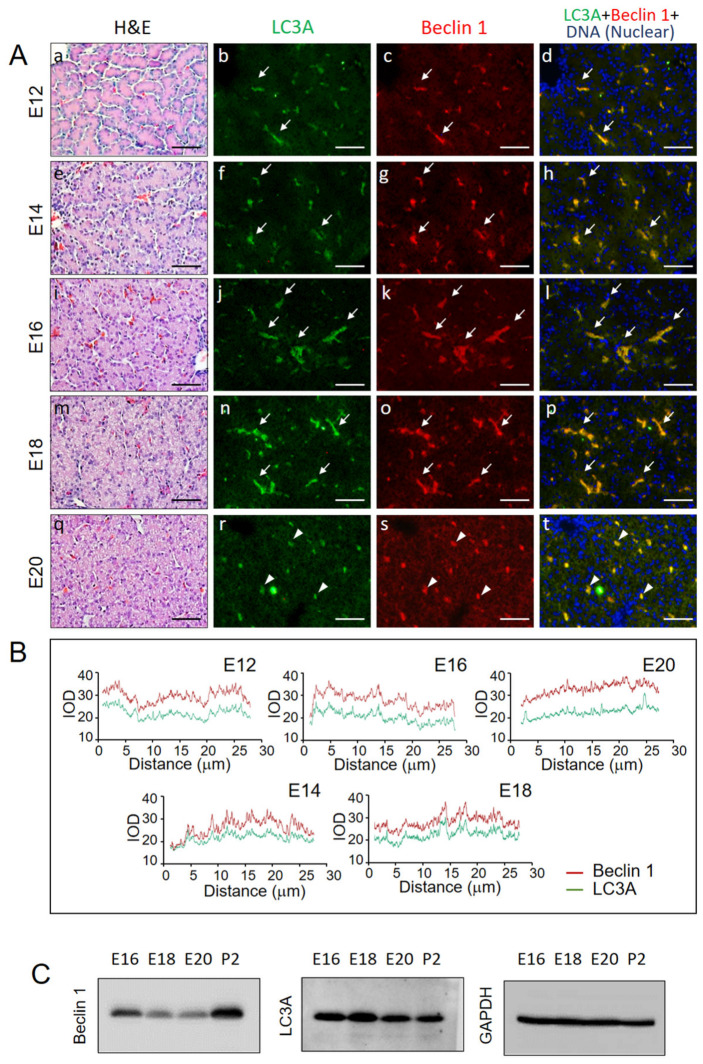
Distribution of LC3A and Beclin 1 in Taihe embryonic livers at different stages of embryonic development. Immuno-fluorescence of LC3A and Beclin 1 in Taihe embryonic livers at E12, E14, E16, E18 and E20 exhibits structural co-localization (Panel (**A**,**B**). (Panels (**A**) (**a**,**e**,**i**,**m**,**q**)) are HE histological view of embryonic livers. Immunofluorescence stainings of LC3A and Beclin 1 are exhibited in Panels (**b**), (**f**), (**j**), and (**n**) and (**r**), and (**c**), (**g**), (**k**), (**o**) and (**s**) respectively. Panels (**d**,**h**,**l**,**p**,**t**) are overlap of LC3A and Beclin 1 with counter staining of Hoechst 33342 on nuclei in the same field of livers at different stages. The structural co-localization of LC3A and Beclin 1 are recorded in detail Panel (**B**) in Taihe embryonic livers at E12, E14, E16, E18 and E20. Expression of Beclin 1 and LC3A proteins in the livers at E16, E18, E20 and P2 were identified using Western blot analysis. Scale bars are 50 μm in panels Aa-t. In Panel (**C**), relative expression levels of Beclin 1 and LC3A proteins were analyzed in liver at different stages of chick embryo development (E16, E18, E20 and P2) by Western blot analysis.

Using immune-blotting analysis and immunofluorescent histochemistry, we focused on the expression of Beclin 1 and LC3A during Taihe liver development against mitochondrial GAPDH control at E16, E18, E20 and sP2 (Figure 6C). Both Beclin 1 and LC3A were distributed in developing livers (Figure 6A) with significant linear co-localization of diverse lengths (Figure 6A(d,h,l,p,t),B). These linear forms showed areas of intersection (Figure 6, arrows). These critical intersections enlarged and broadened during development of hepatic cords and coincided with increase in Beclin 1 and LC3A fluorescence during E16-E20 (Figure 6A). Once the chick hatched and liver development concluded, these linear intersections began breaking up into shorter zones or dotted lines. By P2, Beclin 1 and LC3A activity had lessened to small focal areas (Figure 6A(q–t)).

**Figure 7:**
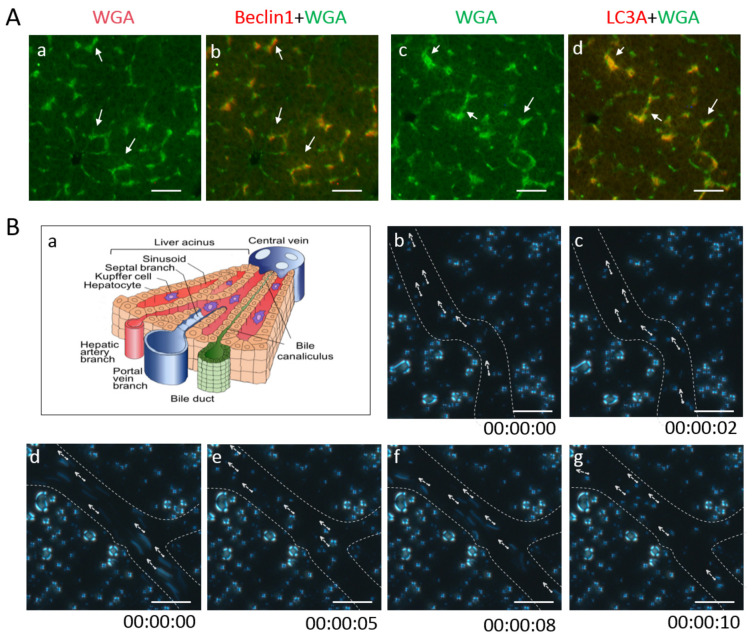
A Time-lapse recording of HLCD flow in sinusoid of Taihe embryonic livers at stage of development E18 under crossed polarized lights. In Panel (**A**), immunofluorescence staining of LC3A (Panel (**A**) (**d**)) and Beclin 1 (Panel **A** (**b**)) co-localize with WGA immunofluorescence (Panel (**A**) (**a**,**c**)), which indicates microstructure of livers with staining on sinusoid wall via indicating cytoplasmic membrane glycoproteins. In Panel (**B**), two sets of the time recordings of LCLD flows are observed between branches of sinusoid/vein branches representative with (Panels (**B**) (**b**–**g**)) following histological structure of liver showed in (Panel (**B**) (**a**)). Scale bars are 50 μm in (Panels (**A**) (**a**–**d**),(**B**) (**b**–**g**)).

We further studied the association of Beclin 1 and LC3A with in vitro liver architecture. Lectin-conjugated wheat germ agglutinin (WGA), which has a high affinity for cell membrane components sialic acid and N-acetylglucosamine glycoproteins moieties, was used to label cytoplasmic membrane. Beclin 1 and LC3A were also co-stained to demonstrate cytoplasmic distribution and colocalization of these proteins. Co-staining of WGA, Beclin 1, and LC3A demonstrated colocalization of Beclin 1 and LC3A to the plasma membrane (Figure 7A(a,b) arrows). Staining for all three was localized around hepatic sinusoids. As we described above, HLCDs were localized in the hepatocytes of hepatic cords in the developing liver. Furthermore, the shape of these HLCDs is variable. HLCDs found in the sinusoids (Figure 7B, dotted lines) are long (Figure 7B, arrow), while those within hepatocytes remain mostly circular (Figure 7B(b–g)), exhibiting that free HLCDs were able to flow in hepatic sinusoids, indicated with round-end arrows (Figure 7A). The close architectural association of HLCDs with cell membrane and autophagy proteins, Beclin and LC3A, leads us to hypothesize that these autophagy proteins play a role in regulating the membrane lipids (cholesterol, cholesterol ester and lecithin) that make up HLCDs.

### 3.7. HLCDs Associated with Beclin 1 and LC3A Share Characteristics with HLCD Found in NAFLD Patients

Many pathophysiological states in the liver have abnormal reactivation of embryonic cell signaling pathways (Table S1). This holds true for NAFLD [8]. As we have shown, the embryonic hepatocytes contain abundant liquid droplets. These embryonic HLCDs have the same composition (cholesterol, cholesterol ester and lecithin) and characteristics (liquid crystalline nature) of fatty droplets found in patient NAFLD liver cells [8]. However, alterations in autophagy gene expression of NAFLD patient livers are unknown.

**Figure 8:**
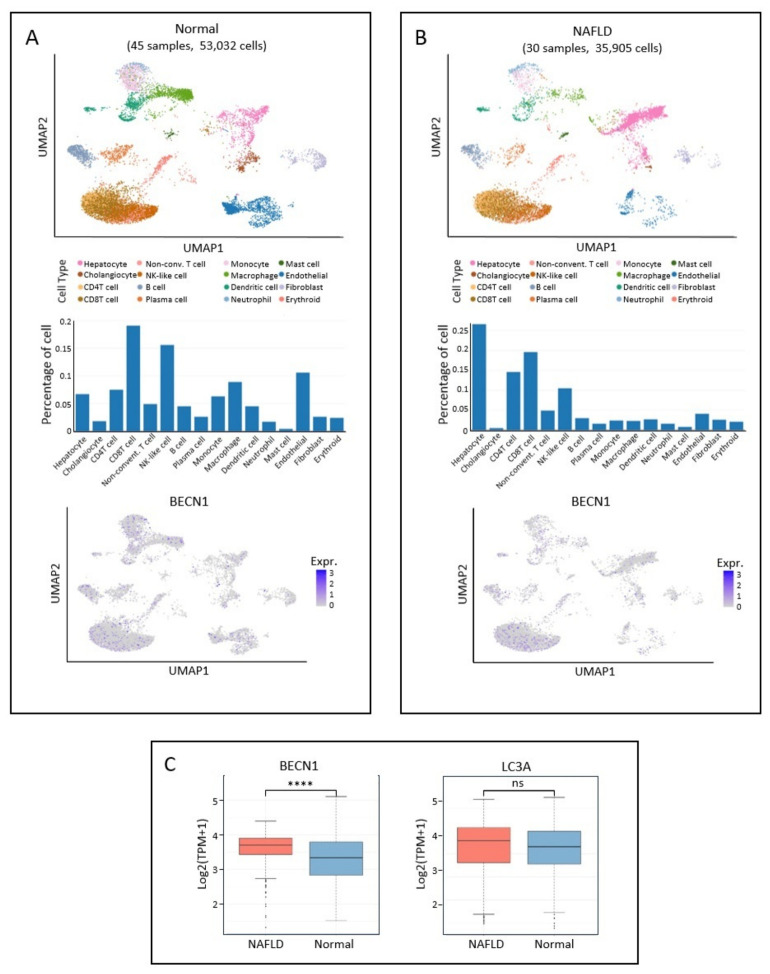
Expression of BECN1 and LC3A in NAFLD or normal tissue according to GepLiver databases accessed on 18 January 2026. Panel (**C**). The differences in BECN1 or MAP1LC3A mRNA expression between NAFLD and normal tissues analyzed using the GepLiver database (**** *p* < 0.0001; ns, no significance) (Panel (**A**)). scRNA-seq analysis on normal liver. UMAP plot (top) shows single-cell aggregation on normal liver tissue. Chart in middle shows the percentage of various cells out of all cells. The bottom shows the UMPA plot showing BECN1 expression levels in different cells (Panel (**B**)). scRNA-seq analysis of normal liver. UMAP plot (top) shows single-cell aggregation on NAFLD tissue. Chart in middle shows the percentage of different cells out of all cells. The bottom shows the UMPA plot showing BECN1 expression levels in various cells.

Using the GepLiver database (http://www.gepliver.org/, accessed on 8 January 2026), we analyzed scRNA-seq data from 53,032 cells across 45 samples using principal component analysis (PCA) for dimensionality reduction and visualized cell relationships with UMAP. This analysis divided the cells into 16 clusters: Hepatocyte, Cholangiocyte, CD4 T cell, CD8 T cell, Non-conventional T cell, NK-like cell, B cell, Plasma cell, Monocyte, Macrophage, Dendritic cell, Neutrophil, Mast cell, Endothelial cell, and Fibroblast (top of Figure 8A,B). Feature genes defining each cluster are listed in Supplementary Table S1. We then analyzed the percentage of each cell type relative to the total number of cells. Compared with normal tissues, NAFLD tissues showed a significant increase in hepatocytes, rising from 6.7% to 26.6%, while monocytes, macrophages, and endothelial cells decreased significantly, from 6.3%, 8.9%, and 10.6% to 2.4%, 2.3%, and 4.1%, respectively. In contrast, fibroblasts and erythroid cells did not exhibit significant changes in NAFLD relative to the other fetal cell types analyzed in relation to HLCD during liver development. Most significantly, we found that human NAFLD tissues exhibited significantly increased BECN1 expression. In this analysis of over 500 NAFLD patient livers, BECN1 expression was markedly elevated in NAFLD tissues compared with normal liver (*n* = 362; *p* < 0.0001). Although LC3A expression in NAFLD tissues were slightly higher than normal tissues, it was not statistically significant (Figure 8A).

**Figure 9:**
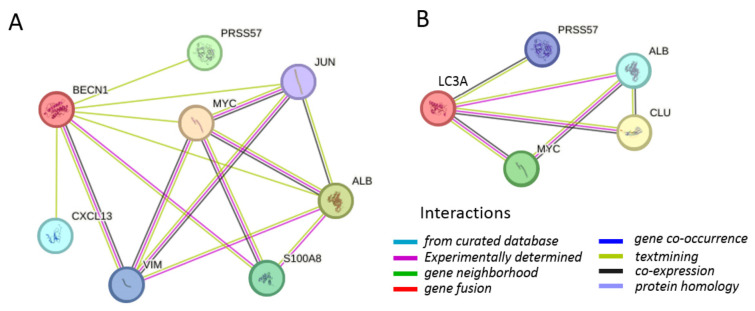
Identification and construction of PPI networks for BECN1 and LC3A accessed on 18 January 2026. Protein–protein interaction (PPI) analysis of BECN1 and LC3A was performed using fetal cell feature genes from the GepLiver database. Direct interactors were identified to construct the PPI networks shown. Panel (**A**) shows the protein–protein interaction (PPI) network of BECN1 direct interactors. Panel (**B**) shows the protein-protein interaction (PPI) network of MAP1LC3A direct interactors.

To determine whether this potentially pathogenic signature reflects gene expression patterns seen during embryonic liver development, we analyzed protein–protein interactions (PPIs) in NAFLD-related fetal cells. Using fetal cell feature genes from the GepLiver database, we constructed PPI networks centered on BECN1 and LC3A. We focused on cell types that showed significant proportional changes in NAFLD compared with normal tissue and identified their direct protein interactors. The BECN1 network included MYC, ALB, PRSS57, S100A8, CXCL13, VIM, and JUN, while the LC3A network included CLU, MYC, ALB, and PRSS57 (Figure 9; Table 2).

In summary, our findings suggest phase transition/separation of HLCDs may share common molecular and cellular mechanisms between embryonic development and NAFLD pathology. This phenomenon may offer a unique perspective for developing potential liquid-crystal-associated strategies for t.

## 4. Discussion

Phase separation and phase transitions have been increasingly recognized as important cellular organization mechanisms for both normal and pathogenic biological processes. Some investigators have suggested these phenomena may simply represent byproducts of molecular interactions [1]. In contrast, our findings support a model in which HLCDs function as developmentally regulated lipid reservoirs that facilitate liver organogenesis. Rather than representing passive accumulations of lipids or incidental byproducts of cellular metabolism, HLCDs appear to be highly organized structures that emerge at a specific developmental stage (E12), undergo coordinated phase transitions (E16-E20), and disappear once liver maturation is largely complete (P2). Their tightly regulated temporal pattern strongly suggests a functional role during embryonic development.

The composition of HLCDs—cholesterol, cholesterol esters, and lecithin—are also essential structural components of cellular membranes. During the period of rapid liver growth, hepatocytes and hepatic progenitor cells must generate large quantities of membrane material to support cell proliferation, differentiation, and tissue remodeling. The transient accumulation of HLCDs beginning at embryonic day 12 may therefore provide a readily available reservoir of membrane lipids that can be mobilized to meet these developmental demands. In this context, HLCDs may serve as specialized storage compartments that coordinate lipid availability with the requirements of liver morphogenesis. Such organization could facilitate efficient storage, trafficking, and mobilization of lipids during periods of rapid tissue expansion. The disappearance of HLCDs during postnatal development is consistent with this interpretation, as the demand for extensive membrane synthesis decreases once organ architecture has been established.

A particularly intriguing aspect of HLCD biology is their dynamic phase behavior. We observed both embryonic phase transitions, characterized by the widespread appearance of HLCDs throughout the liver, and intra-droplet phase transitions, in which individual droplets convert from non-birefringent isotropic structures to birefringent anisotropic liquid crystal states. These transitions occur in a highly reproducible developmental sequence, suggesting that they are regulated processes rather than spontaneous physical events. Similar phase-transition phenomena have been described in protein condensates, nucleoli, and other biomolecular assemblies, where changes in physical state contribute directly to biological function. Our observations extend this concept to lipid-rich structures and suggest that liquid crystal organization may represent an additional mechanism by which cells regulate developmental processes. Furthermore, our data suggests that autophagy proteins Beclin 1 and LC3A, and thus autophagosomes, may play a role in the regulation of HLCD lipids.

Increasing evidence indicates that phase separation and phase transitions are fundamental mechanisms governing a wide range of cellular processes. In animal models, the onset and progression of NAFLD coincide with the appearance and dramatic accumulation of cholesteryl oleate-rich liquid crystal droplets. Observations in NAFLD patients further suggest that liquid crystallization may be associated with lipid phase transitions into liquid-crystalline states [8]. Similarly, Lee and colleagues reported significant enrichment of cholesteryl esters during prostate carcinogenesis, implicating altered lipid metabolism in oncogenic processes [54,55]. In amyotrophic lateral sclerosis (ALS), mutations associated with age-related neurodegenerative diseases accelerate the liquid-to-solid phase transition of the ALS-associated protein FUS. This promotes the formation of aberrant aggregates linked to cellular dysfunction [56,57]. Protein phase separation under physiological conditions is influenced by factors such as salt concentration and arginine methylation, suggesting a novel biophysical framework for understanding neurodegenerative diseases, including frontotemporal dementia (FTD) and ALS [11,12]. In vitro studies by George-Hyslop and colleagues further showed that FUS-mediated phase separation is tightly regulated by factors such as arginine methylation and the molecular chaperone ADMA, which influence the progression from dispersed molecules to liquid droplets, hydrogels, and fibrillar assemblies [56,57]. This highlights the importance of understanding how cells control the assembly, material properties, and dissolution of biomolecular condensates and this may assist in our understanding, and thus treatment, of human diseases.

## 5. Conclusions

Taken together, our findings demonstrate that embryonic liver development is accompanied by both large-scale phase transitions and intra-droplet phase transitions that generate abundant HLCDs. The temporal regulation, characteristic composition, and conserved occurrence of these structures suggest that they play important biological roles rather than representing passive byproducts of lipid metabolism. The concurrent timing of their appearance and phase changes with autophagy proteins, Beclin 1 and LC3A, suggest autophagy may play a role in HLCDs regulation through autophagy-related storage and breakdown of cell membrane components. Future studies should focus on confirming the molecular regulators governing HLCD formation and phase behavior, as well as determining how these processes contribute to liver organogenesis and disease susceptibility. Understanding the mechanisms that control liquid crystal organization in embryonic tissues may provide new insights into both developmental biology and the pathogenesis of lipid-associated diseases.

## Figures and Tables

**Table 1:** X-ray diffraction pattern of liquid crystal and crystal and their extract of embryonic livers.

Standard Cholesteryl Oleate		E20 *		E20 **		E17 **		Standard Cholesterol	
d(Å)	I/I_0_	d(Å)	I/I_0_	d(Å)	I/I_0_	d(Å)	I/I_0_	d(Å)	I/I_0_
11.01	9.9								
		10.65	16.5						
10.3	7.4								
		9.778	30.6						
9.442	44.1								
								9.396	31.1
								8.411	43.3
8.301	8.4	8.336	11.8						
								7.925	26.2
7.86	16.8	7.796	16.1						
								7.642	26.2
								7.464	21.3
7.35	7.4								
7.054	7.9								
		6.952	12.5					6.953	51.2
6.618	6.4	6.681	11.0						
								6.437	47.6
								6.336	44.5
								6.154	32.9
6.001	17.8	5.964	24.7						
				5.894	54.5				
								5.782	85.4
								5.719	86.0
5.717	7.4								
		5.666	14.5						
5.474	11.4	5.489	14.5						
		5.294	17.6					5.287	100
								5.257	98.8
5.195	10.9			5.164	68.6	5.199	83.8		
		5.126	20.0					5.138	70.1
				4.981	61.5			4.937	57.9
4.918	100	4.918	100	4.887	100	4.934	100		
4.704	16.8			4.744	59.6			4.763	37.8
								4.636	35.4
4.604	17.3	4.603	32.2	4.563	82.7	4.603	82.3		
		4.476	18.8	4.432	65.4				
4.317	8.4								
								4.274	38.4
								4.23	36.6
4.057	48.0	4.09	44.7	4.062	71.2	4.104	88.2		
3.903	12.4	3.942	16.1						
								3.805	26.2
				3.735	64.1				
		3.581	15.3					3.583	26.2
3.48	10.4	3.486	13.3						
		3.234	13.7						
3.136	8.4								

Note: * indicates extracts from liver at E20; **, cryopreserved livers.

**Table 2:** Comparison analysis on Beclin 1 and LC3A-assoicated lineages and the feature gene with high cluster-resolution in liver between normal and NAFLD.

#	Labels	Cell_Type/Lineage	Feature_Genes	Gene Functions	Refs.
1	Fetal Erythroid	Fetal_Erythroid_02_SYNGR1	MYC	Myc proto-oncogene protein: Transcription factor binding to DNA in a non-specific manner, and specifically recognizing the core sequence 5′-CAC[GA]TG-3′; Activating the transcription of growth-related genes via binding to the VEGFA promoter, promoting VEGFA production and subsequent sprouting angiogenesis	[34,35,36]
2	Fetal Hepatocyte	Fetal_Hepatocyte_01_ALB	ALB	Serum albumin: the main protein of plasma strong binding capacity for water, Ca^(2+)^, Na^(+)^, K^(+)^, fatty acids, hormones, bilirubin and some drugs; regulating the colloidal osmotic pressure of blood. Zinc transporter plasma zinc (~80%), major calcium and magnesium transporter (~45%); a non-specific manner calcium binding	[37,38,39]
3	Fetal HSC/MPP	Fetal_HSC/MPP_01_SPINK2	PRSS57	Serine protease 57; Serine protease that cleaves preferentially after Arg residues. Can also cleave after citrulline and methylarginine residues; Belongs to the peptidase S1 family	[40,41]
4	Fetal Monocyte	Fetal_Mo_01_S100A9	S100A8	Protein S100-A8: calcium- and zinc-binding protein regulating inflammatory processes and immune response; inducing neutrophil chemotaxis and adhesion; Intraellular role, facilitating leukocyte arachidonic acid trafficking and metabolism, modulation of the tubulin-dependent cytoskeleton during migration of phagocytes and activation of the neutrophilic NADPH- oxidase; Extracellular roles by activating NADPH-oxidase-the cell membrane axis, transferring arachidonic acid, binding to NCF2/P67PHOX, involving pro-inflammatory, antimicrobial, oxidant-scavenging and apoptosis-inducing activities via mitochondria and lysosome-mediated autophagy and apoptosis via reacting ROS involving BNIP3	[42,43,44]
5	Fetal Neutrophil-Myeloid Progenitor Cell	Fetal_Neut-Mye-Prog_01_MPO	S100A8	Similar to the above	[42,43,44]
6	Fetal T Cell	Fetal_Tcell_01_CD8A	CXCL13	C-X-C motif chemokine 13: Chemotactic for B-lymphocytes but not for T-lymphocytes, monocytes and neutrophils. Does not induce calcium release in B- lymphocytes; Binding to BLR1/CXCR5	[45,46,47]
			JUN	Transcription factor AP-1:Recognizing and binding to the enhancer 5′-TGA[CG]TCA-3′; Promoting NR5A1 when phosphorylated by HIPK3; Leading increased steroidogenic gene expression via cAMP pathway stimulation; Involving activated KRAS-mediated USP28 binding to the USP28 promoter in CRC cells. Member of Jun subfamily	[48,49,50,51]
7	Fetal T Cell	Fetal_Tcell_02_IL7R	VIM	Vimentin; Vimentins are class-III intermediate filaments found in various non-epithelial cells, especially mesenchymal cells. Vimentin is attached to the nucleus, endoplasmic reticulum, and mitochondria, either laterally or terminally	[48,52,53]

Abbreviations: Myc, Myc proto-oncogene protein; ALB, Albumin; PRSS57, Serine protease 57; S100A8, S100 Calcium Binding Protein A8; CXCL13, C-X-C Motif Chemokine Ligand 13; VIM, Vimentin; JUN, Transcription factor AP-1; CRC, colorectal cancer. Detailed information, the GepLiver database (http://www.gepliver.org/; accessed on 15 January 2026).

## Data Availability

The data that support the findings of the study regarding this manuscript are available after the corresponding author’s approval upon reasonable request.

## Supplementary Materials

The following supporting information can be downloaded at: https://www.mdpi.com/article/10.3390/biology15141168/s1, Figure S1: Fluidity of hepatic birefringent lipid droplets in Taihe embryonic livers at E18; Figure S2: Time-lapse recording on fluidity of Taihe hepatic birefringent droplets in process of fusion of two HLCD Maltese crosses; Figure S3: In vitro thermal phase transitions of Taihe hepatic birefringent droplets as Maltese crosses from anisotropic to isotropic state, and resumed from isotropic to Maltese crosses; Figure S4: Originals for Figure 5 Examination of protein expression of autophagy related genes; Table S1: Fetal developing related cell types or lineages involved in adult including in normal and NAFLD livers.

## Abbreviations

The following abbreviations are used in this manuscript:

ADMA	asymmetric dimethylarginine
ALS	amyotrophic lateral sclerosis
ani-HLCD	anisotropic hepatic liquid crystal droplets
CD51	Integrin αV
DIC	differential interference contrast
DS	divergence slit
E	embryo
EB	embryoid body
ECM	extracellular matrix
FKBP	FK506 binding protein
FITC	Fluorescein isothiocyanate
FTD	frontotemporal dementia
FUS	fused in sarcoma
GAPDH	Glyceraldehyde-3-phosphate dehydrogenase
H&E	Hematoxylin and Eosin
HLC	hepatic liquid crystal
HLCD	hepatic liquid crystal droplet
HLCDs	hepatic liquid crystal droplets
HLD	hepatic lipid droplet
HRP	horseradish peroxidase
hSC	human stem H1 cells
iso-HLD	isotropic hepatic lipid droplets
iso-LD	isotropic lipid droplet
LC	liquid crystal
LDAH	lipid droplet associated hydrolase
MAM	mitochondria-associated ER membranes
MAP	microtubule-associated protein
MAP1LC3A/LC3A	microtubule-associated protein 1 light chain 3 alpha
MC	Maltese cross
MCs	Maltese crosses
MMP	Matrix metalloproteinase
NAFLD	non-alcoholic fatty liver disease
OCT	optimum cutting temperature
P	postnatal
P granules	protein granules
P&R	pressure and release
PBS	phosphate buffered saline
PBS-T	phosphate-buffered saline-Tween 20
PE	phosphatidylethanolamine
PFA	paraformaldehyde
Ph_iso→aniso_	phase transition_isotropic→anisotropic_
RIPA	radioimmunoprecipitation assay
RNP	RNA-protein complex
RRX	Rhodamine Red™-X
SAXS	small-angle X-ray scattering
SOAT1	sterol O-acyltransferase 1
Taihe fowl	Taihe black-bone silky fowl
TLC	thin-layer chromatography
WGA	wheat germ agglutinin
XRD	X-ray Diffraction
