# Supplementary material for: Embryonic Phase Transition/Separation on Hepatic Liquid Crystal Droplets Is Essential for Liver Development
Source: Biology (Basel). 2026 Jul 16;15(14):1168. doi: 10.3390/biology15141168 (PMC13403541; doi:10.3390/biology15141168)

Originals for

Figure 5. Examination of protein expression of autophagy related genes

Detail information included in *Materials and Methods*

Merge, Marker and Chemo-luminescence (30s)

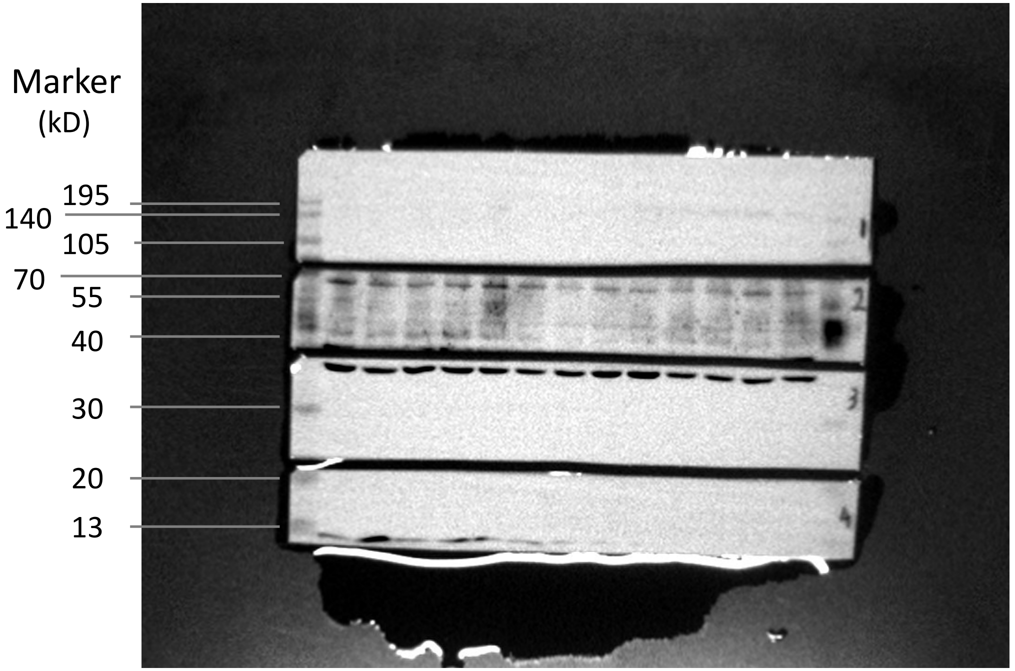

10% SDS-PAGE

Chemo-luminescence (Exposure 60s)

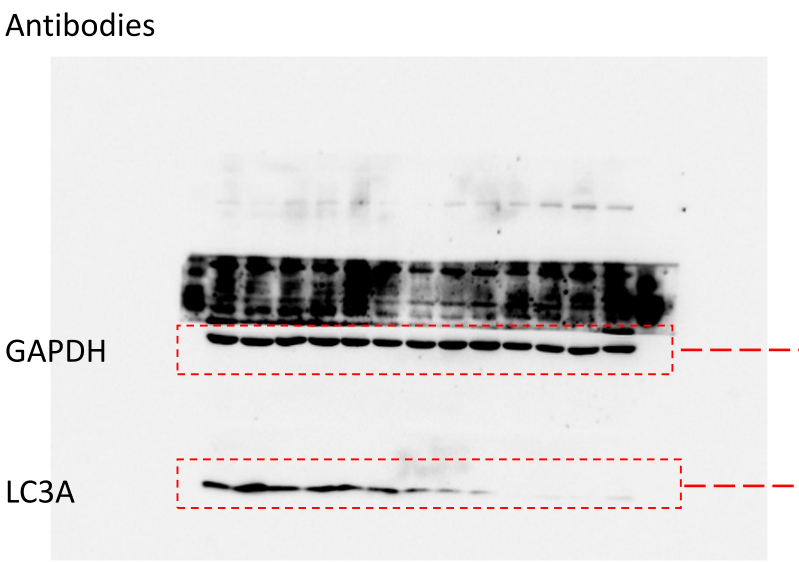

Figure 5

Panel A – GAPDH

Panel A – LC3A

Originals for

Figure 5. Examination of protein expression of autophagy related genes

Merge, Marker and Chemo-luminescence (30s)

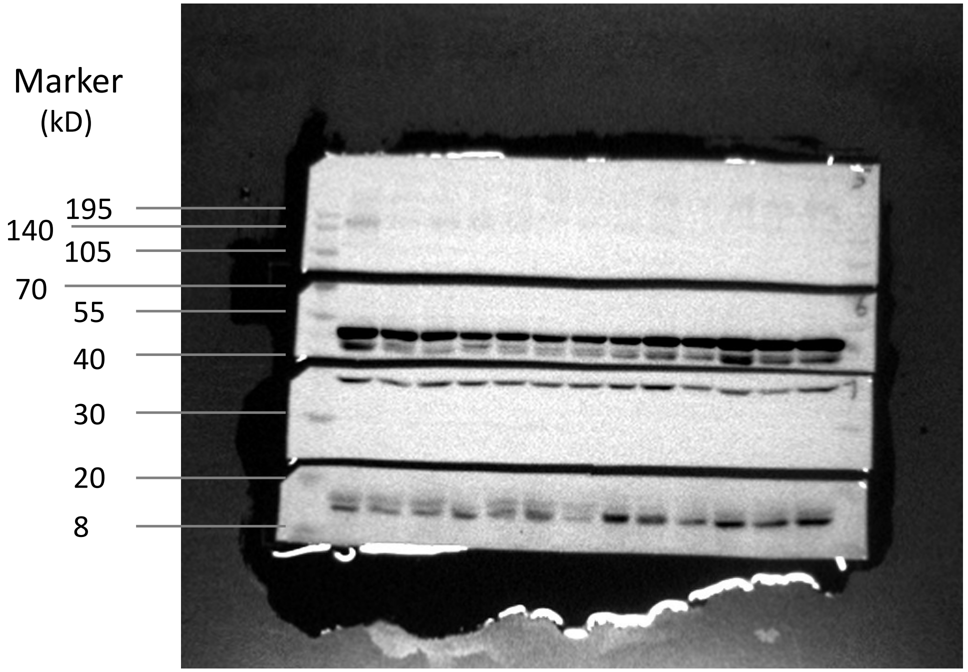

10% SDS-PAGE

Detail information included in *Materials and Methods*

Figure 5

Chemo-luminescence (Exposure 30s)

Chemo-luminescence (Exposure 60s)

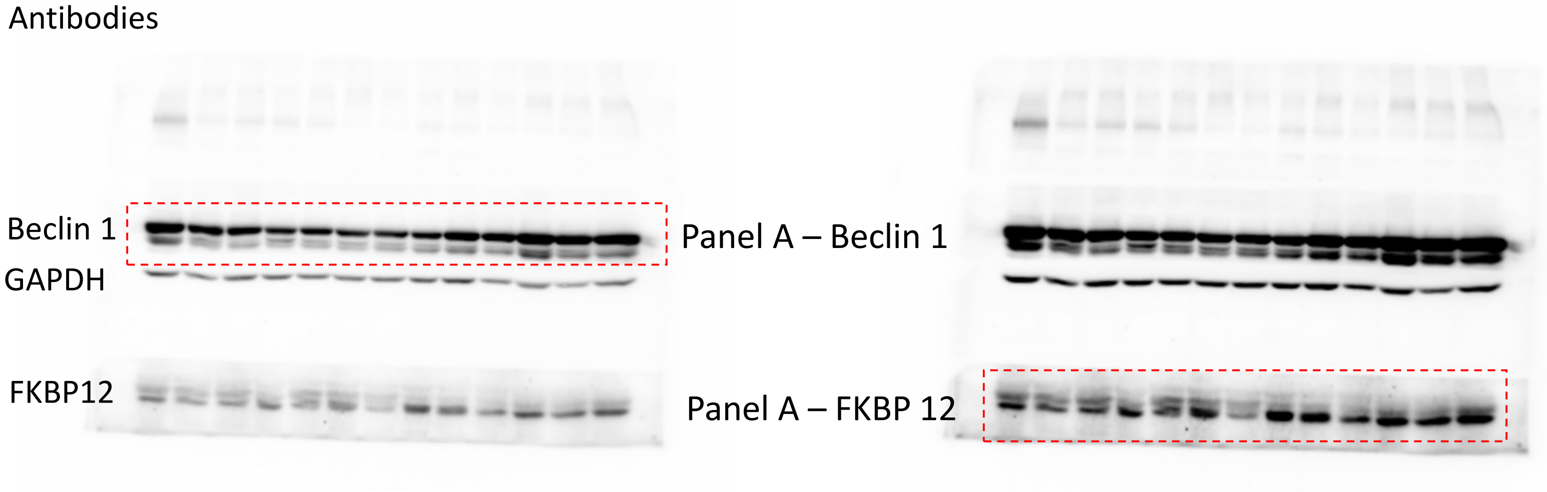

Detail information included in Materials and Methods

Originals for

Figure 5. Examination of protein expression of autophagy related genes

Detail information included in *Materials and Methods*

Merge, Marker and Chemo-luminescence (60s)

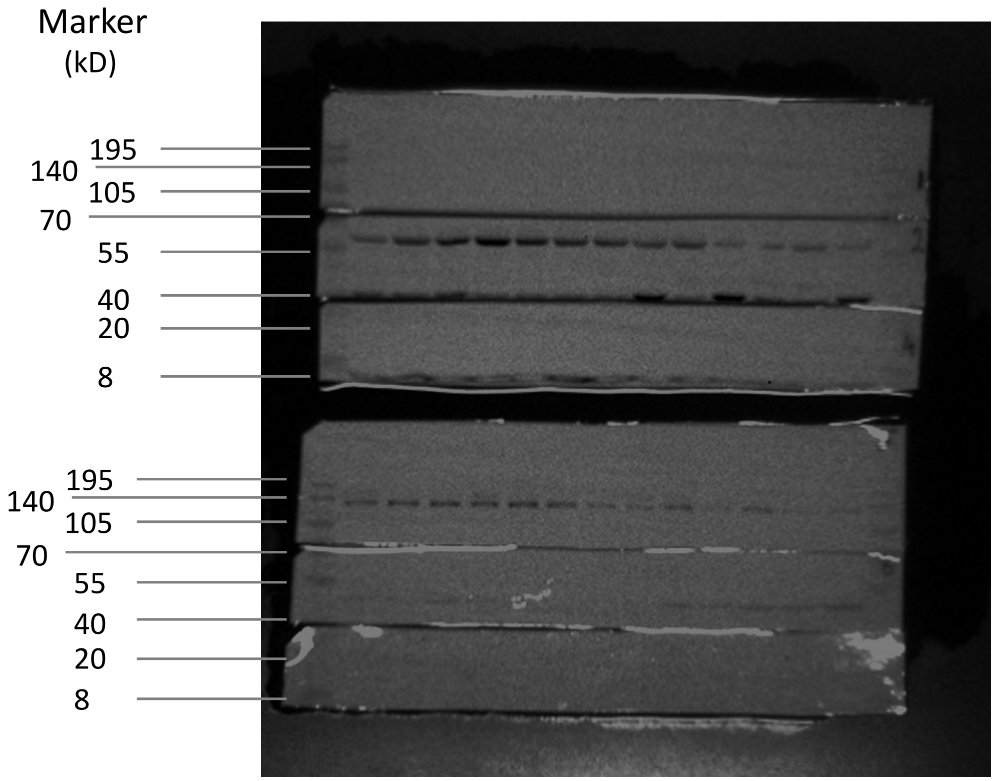

10% SDS-PAGE

Chemo-luminescence (Exposure 60s)

Figure 5

Antibodies

FKBP52

Integrin αV

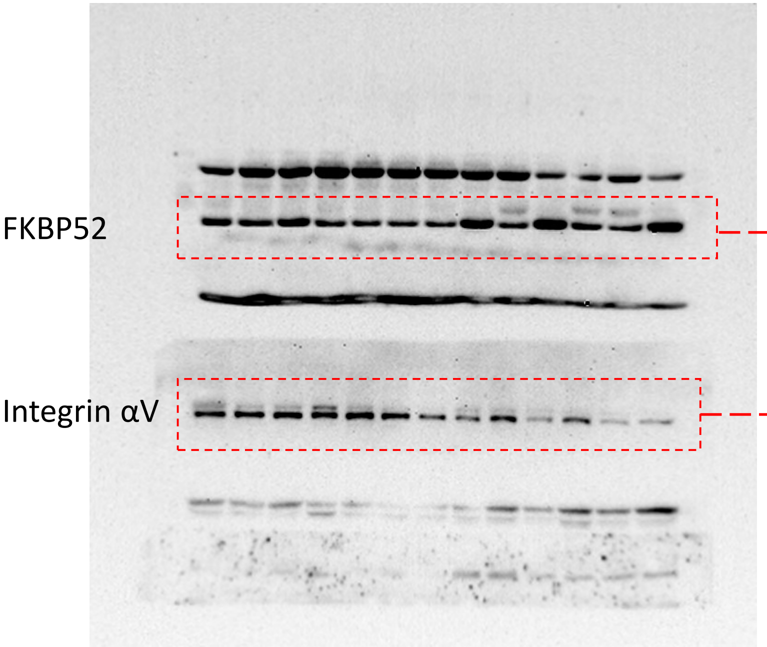

Panel A – FKBP 52

Panel A – Integrin αV

Originals for

Figure 5. Examination of protein expression of autophagy related genes

Detail information included in *Materials and Methods*

Figure 5

Merge, Marker and Chemo-luminescence (30s)

Marker (kD)

195
140
105
70
55
40
30
20

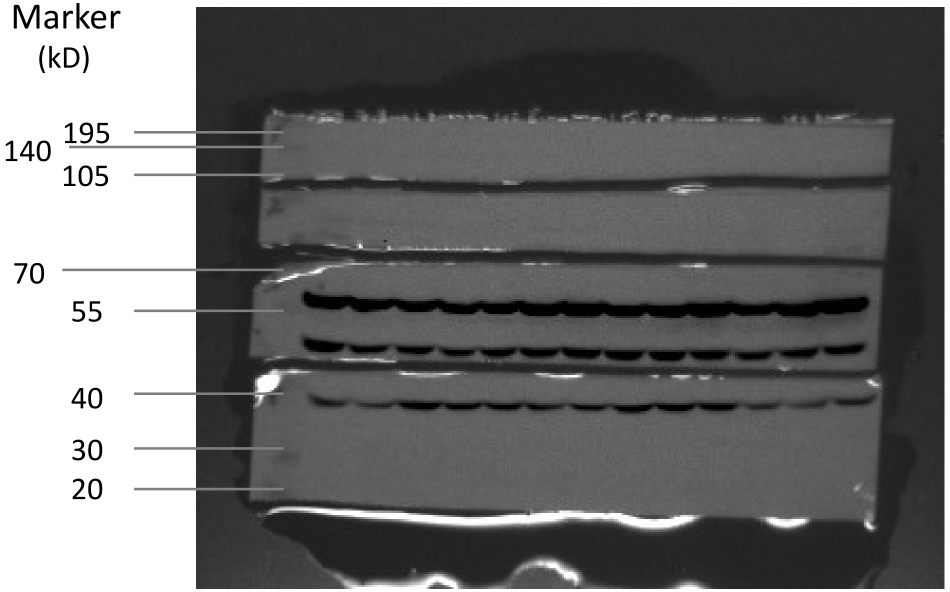

8% SDS-PAGE

Chemo-luminescence (Exposure 15s)

Antibodies

MMP 8

GAPDH

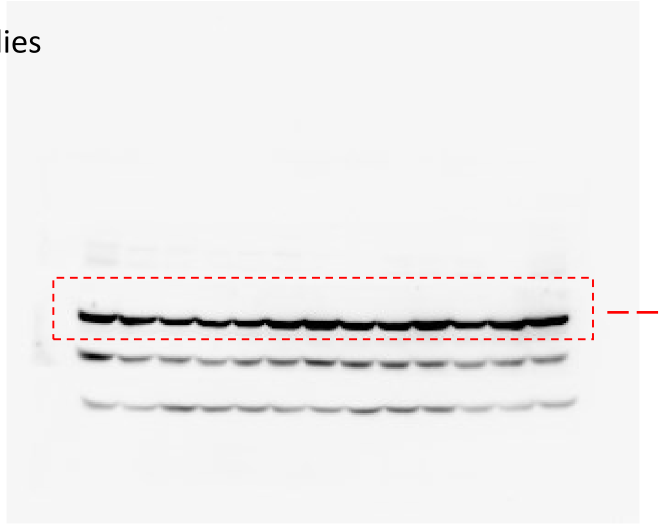

Panel A – MMP 8

Originals for

Figure 5. Examination of protein expression of autophagy related genes

Detail information included in *Materials and Methods*

Figure 5

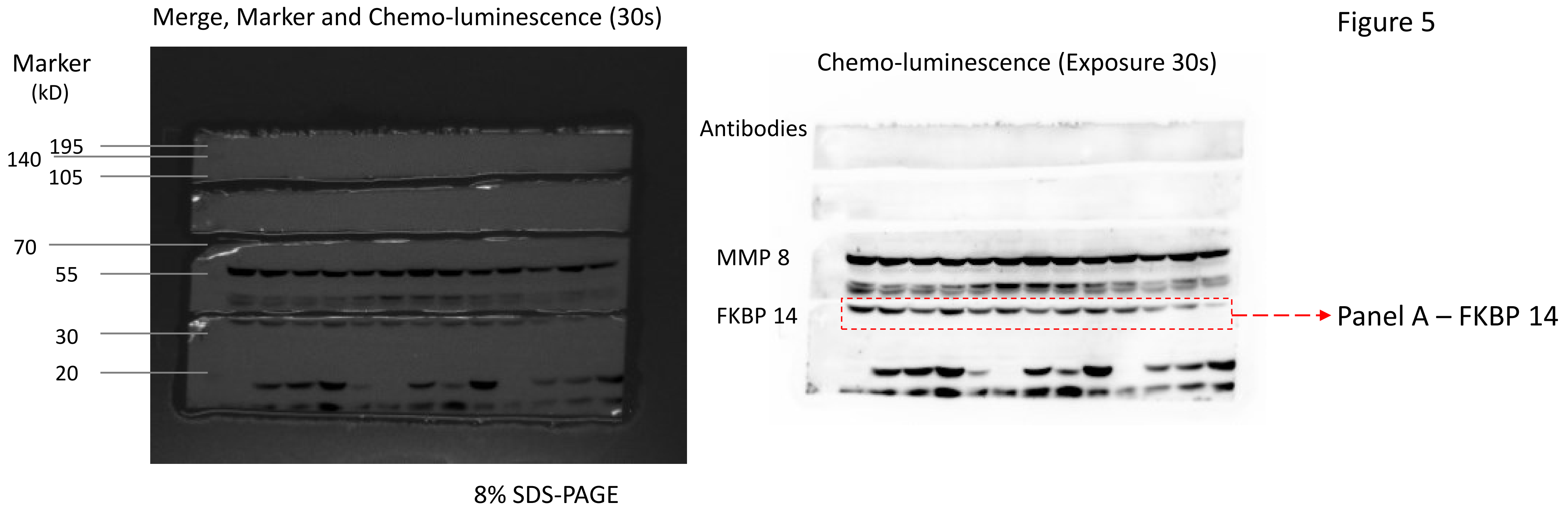

Originals for

Figure 5. Examination of protein expression of autophagy related genes

Detail information included in *Materials and Methods*

Figure 5

Merge, Marker and Chemo-luminescence (60s)

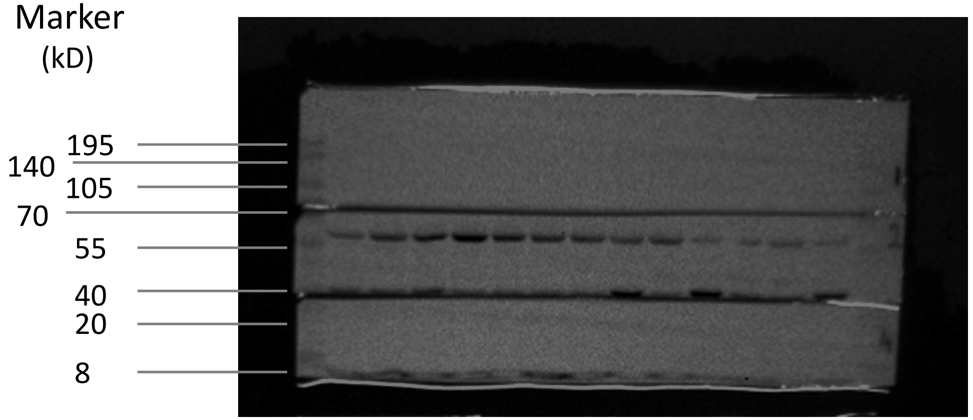

10% SDS-PAGE

Chemo-luminescence (Exposure 5s)

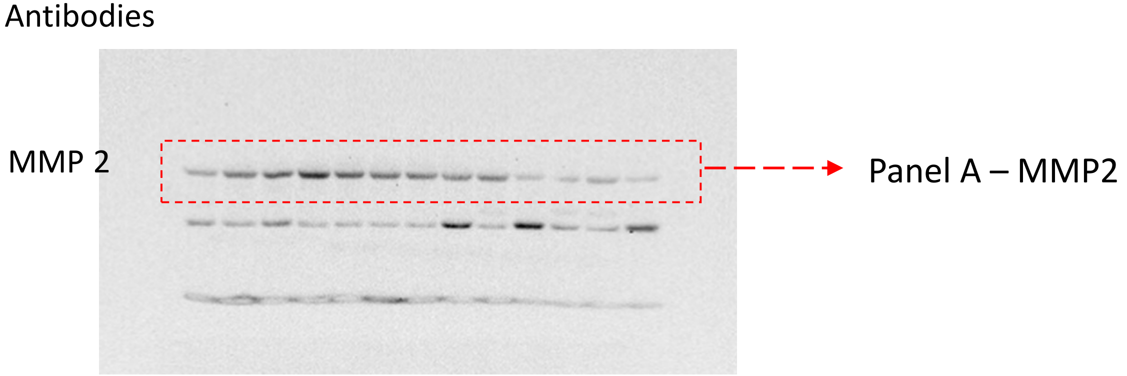

Supplement: Supplementary file 1 [file biology-15-01168-s001.zip › Figure-S4.pdf]
