# Supplementary material for: Embryonic Phase Transition/Separation on Hepatic Liquid Crystal Droplets Is Essential for Liver Development
Source: Biology (Basel). 2026 Jul 16;15(14):1168. doi: 10.3390/biology15141168 (PMC13403541; doi:10.3390/biology15141168)
Supplement: Supplementary file 1 [file biology-15-01168-s001.zip › Table-S1.pdf]

Table S1 Fetal developing related cell types or Lineages involved in adult including in normal and NAFLD livers

#	Cell_Type/Lineage	Cluster_ Resolution	Feature_Genes	Suggested_Labels	Freq_All	Freq_Fetal	Freq_Normal	Freq_NAFLD
1	Fetal_Cholangiocyte_01_SPP1	High	FDCSP;SPP1;CLU;TM4SF4;ANXA4;S100A2;KRT17;KRT8;CD24;KRT19	Fetal Cholangiocyte	0.001	0.009	0	NA
2	Fetal_Endo_01_FCN3	High	FCN3;DNASE1L3;CCL14;CRHBP;CLEC1B;NTS;RAMP2;S100A16;OIT3;ACP5	Fetal Endothelial Cell	0.005	0.093	0	NA
3	Fetal_Endo_02_PLVAP	High	CRHBP;DAB2;HMOX1;CLEC1B;ACP5;GNG11;OIT3;RNASE1;RAMP2;S100A16	Fetal Endothelial Cell	0	0.005	NA	NA
4	Fetal_Erythroid_01_GYPA	High	GYPA;HBG2;PRDX2;HEMGN;AHSP;ALAS2;BLVRB;HBG1;HMBS;MYL4	Fetal Erythroid	0.011	0.189	0	0
5	Fetal_Erythroid_02_SYNGR1	High	FAM178B;CA1;HBD;HBB;SYNGR1;TMEM14C;MYC;KCNH2;TMEM14B;CNRIP1	Fetal Erythroid	0.007	0.102	0.002	0
6	Fetal_Erythroid_03_HBA1_MT1H	High	HBE1;HBZ;HBA2;SLC4A1;HBB;HBM;HBA1;ALAS2;HBG1;HBG2	Fetal Erythroid	0.003	0.048	0	NA
7	Fetal_Erythroid_04_HBE1	High	HBE1;HBZ;CDKN1A;HBA1;HBA2;HBG1;DDIT3;GADD45A;SOD1;MAP1B	Fetal Erythroid	0.001	0.011	NA	NA
8	Fetal_Fibr_01_CXCL14	High	CXCL14;TPM1;PTN;MDK;IGFBP3;RBP1;BEX3;KRT18;COLEC11;CRABP2	Fetal Fibroblast	0.003	0.045	NA	NA
9	Fetal_Fibr_02_COL1A1	High	COLEC11;DCN;IGFBP3;COL1A1;COL3A1;SPARC;BGN;COL1A2;PTN;GPX3	Fetal Fibroblast	0.002	0.038	NA	NA
10	Fetal_Fibr_03_KRT19	High	MGP;UPK3B;KRT19;TFPI2;KRT8;KRT18;TNNT1;SPRR2F;OGN;CAV1	Fetal Fibroblast	0.001	0.011	NA	NA
11	Fetal_Hepatoblast_01_SPINK1	High	SPINK1;AFP;APOA2;APOA1;SERPINA1;APOB;TTR;FABP1;MT1E;RBP4	Fetal Hepatoblast	0.004	0.053	NA	NA
12	Fetal_Hepatocyte_01_ALB	High	ALB;APOC3;AHSG;MT1G;APOH;VTN;TTR;MT1H;ALDOB;FABP1	Fetal Hepatocyte	0.003	0.053	0	0
13	Fetal_HSC/MPP_01_SPINK2	High	SPINK2;SMIM24;AIF1;CYTL1;SOX4;CD34;PRSS57;EIF3E;SELL;PLAC8	Fetal HSC/MPP	0.003	0.052	0	0
14	Fetal_Megakaryocyte_01_GATA2	High	TPSB2;PRG2;HPGDS;CPA3;FCER1A;LMO4;TPSAB1;GATA2;CNRIP1;HBD	Fetal Megakaryocyte	0.002	0.039	0	0.001
15	Fetal_Megakaryocyte_02_PF4	High	PPBP;PF4;GP1BB;RGS18;PLEK;CMTM5;GP9;TUBB1;SDPR;NRGN	Fetal Megakaryocyte	0.002	0.031	0	0
16	Fetal_Mo_01_S100A9	High	S100A9;LYZ;S100A8;FCN1;S100A12;RETN;CSTA;MNDA;LGALS1;IFI30	Fetal Monocyte	0.002	0.024	0	0
17	Fetal_Mp_01_C1QB	High	C1QB;C1QA;C1QC;MS4A7;HMOX1;LGMN;CD5L;FTL;SLC40A1;LIPA	Fetal Monocyte	0.003	0.057	0	0
18	Fetal_Neut-Mye-Prog_01_MPO	High	DEFA3;PRTN3;MPO;AZU1;S100A8;LYZ;S100A9;DEFA4;CAMP;LTF	Fetal Neutrophil-Myeloid Progenitor Cell	0.002	0.034	0	0.002
19	Fetal_NK_01_CCL3	High	CCL3;CCL4;KLRB1;XCL1;XCL2;CMC1;NKG7;KLRD1;TRDC;KLRF1	Fetal NK Cell	0.005	0.015	0.009	0.007
20	Fetal_NK_02_GNLY	High	GNLY;GZMB;NKG7;FGFBP2;PRF1;KLRD1;CCL4;CTSW;CST7;FCGR3A	Fetal NK Cell	0.003	0.002	0.005	0.005
21	Fetal_Pre-B_01_IGHM	High	IGHM;IGLL1;TCL1A;CD79B;VPREB3;IGKC;IGLC1;VPREB1;IGLC3;IGLC2	Fetal Pre-B Cell	0.004	0.061	0	0
22	Fetal_Tcell_01_CD8A	High	CD8A;CXCL13;CCL5;DUSP4;CD3D;RGS1;TRAC;TRBC2;IL32;CCL4L2	Fetal T Cell	0.006	0	0.003	0.002
23	Fetal_Tcell_02_IL7R	High	IL7R;ANXA1;CRIP1;VIM;JUN;LTB;TNFAIP3;LMNA;CXCR4;CD52	Fetal T Cell	0.006	0.005	0.002	0.003
24	Fetal_Tcell_03_TIGIT	High	TNFRSF4;IL32;DUSP4;TNFRSF18;TRAC;BATF;TIGIT;IL2RA;CD2;SPOCK2	Fetal T Cell	0.002	0	0	0.001
25	Fetal_Tcell_04_GNLY	High	GNLY;GZMA;KLRC1;TNFRSF18;CD7;CD247;TRDC;HSPA6;IER5;CAPG	Fetal T Cell	0.001	0	0	0
